# Esters of Bendamustine Are by Far More Potent Cytotoxic Agents than the Parent Compound against Human Sarcoma and Carcinoma Cells

**DOI:** 10.1371/journal.pone.0133743

**Published:** 2015-07-21

**Authors:** Stefan Huber, Johannes Philip Huettner, Kristina Hacker, Günther Bernhardt, Jörg König, Armin Buschauer

**Affiliations:** 1 Pharmaceutical and Medicinal Chemistry II, Institute of Pharmacy, University of Regensburg, Regensburg, Germany; 2 Pharmacology and Toxicology, Institute of Pharmacy, University of Regensburg, Regensburg, Germany; 3 Institute of Experimental and Clinical Pharmacology and Toxicology, Friedrich-Alexander-Universität Erlangen-Nürnberg, Erlangen, Germany; University of Hawaii Cancer Center, UNITED STATES

## Abstract

The alkylating agent bendamustine is approved for the treatment of hematopoietic malignancies such as non-Hodgkin lymphoma, chronic lymphocytic leukemia and multiple myeloma. As preliminary data on recently disclosed bendamustine esters suggested increased cytotoxicity, we investigated representative derivatives in more detail. Especially basic esters, which are positively charged under physiological conditions, were in the crystal violet and the MTT assay up to approximately 100 times more effective than bendamustine, paralleled by a higher fraction of early apoptotic cancer cells and increased expression of p53. Analytical studies performed with bendamustine and representative esters revealed pronounced cellular accumulation of the derivatives compared to the parent compound. In particular, the pyrrolidinoethyl ester showed a high enrichment in tumor cells and inhibition of OCT1- and OCT3-mediated transport processes, suggesting organic cation transporters to be involved. However, this hypothesis was not supported by the differential expression of OCT1 (*SLC22A1*) and OCT3 (*SLC22A3*), comparing a panel of human cancer cells. Bendamustine esters proved to be considerably more potent cytotoxic agents than the parent compound against a broad panel of human cancer cell types, including hematologic and solid malignancies (e.g. malignant melanoma, colorectal carcinoma and lung cancer), which are resistant to bendamustine. Interestingly, spontaneously immortalized human keratinocytes, as a model of “normal” cells, were by far less sensitive than tumor cells against the most potent bendamustine esters.

## Introduction

**Fig 1:**
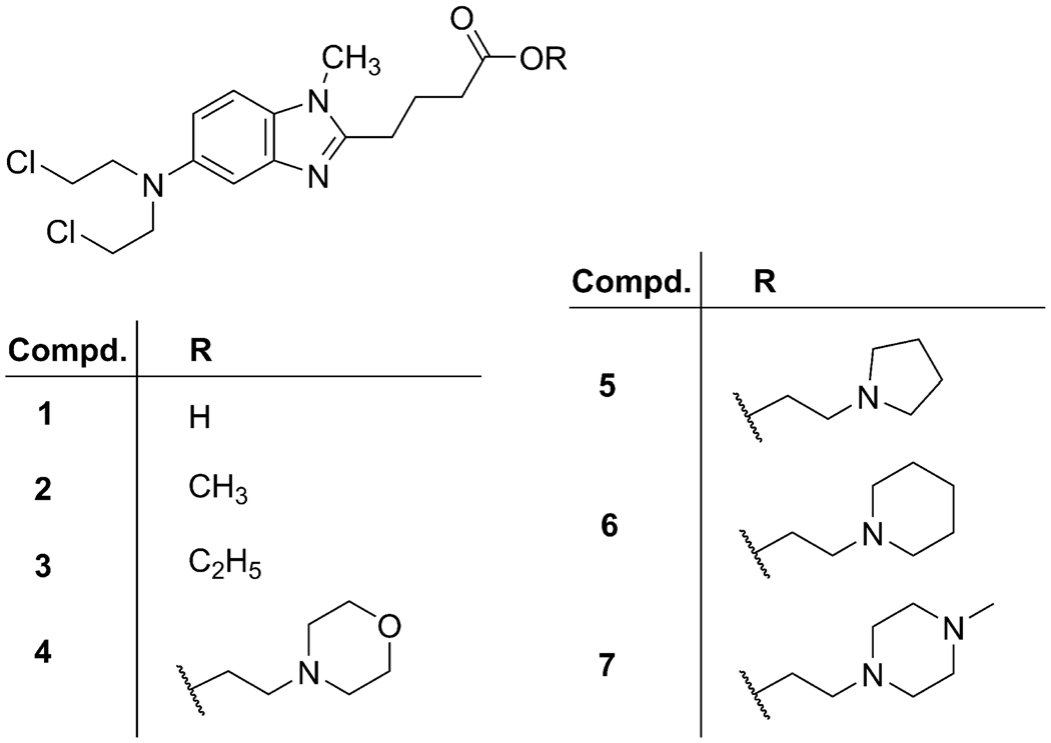
Structures of bendamustine (1) and the bendamustine esters 2–7.

The alkylating agent bendamustine (**1**, Fig 1) was synthesized in 1963 [1, 2] and developed as an anticancer drug in the German Democratic Republic [3–5]. In the 1990s bendamustine got into the focus of research again [6]. It is approved for the treatment of chronic lymphocytic leukemia (CLL) [7, 8], indolent non-Hodgkin lymphoma (NHL) [9] and multiple myeloma (MM) [10, 11] or as second line therapy of refractory diseases [12–14] in various countries. Current clinical trials suggest beneficial effects in the treatment of solid cancer types such as breast cancer [15] or small-cell lung cancer [16]. Most treatment regimens include bendamustine in combination with other anticancer drugs including biologicals [17–20], for instance rituximab [17, 19, 20]. It has been hypothesized that, apart from the alkylating nitrogen mustard group (N-Lost), the benzimidazole scaffold may contribute to the antitumor activity for instance, due to antimetabolite properties, by facilitating nuclear transport or inhibiting DNA repair [6]. Besides the alkylation of DNA, causing strand breaks, bendamustine induces the expression of p53 [21], triggers apoptosis [22] and down-regulates mitotic checkpoints, leading to mitotic catastrophe [23, 24]. Recent patent applications aimed at formulations for oral administration [25–27]. Alkyl (C_1_ –C_24_) esters of bendamustine were reported as potential prodrugs for intravenous application [28], and biodegradable polyphosphoesters were described as an approach to stabilize bendamustine in solution [29]. Another approach aimed at increasing the cytotoxicity by constructing dimeric and dendrimeric bendamustine derivatives [30].

Exploring the properties of derivatives of bendamustine (**1**), esters of bendamustine (**2–7**, Fig 1) [31] comprising basic moieties (**4–7**) were prepared as potential prodrugs with higher solubility compared to simple esters such as compounds **2** and **3**, which were mainly prepared as synthetic intermediates [32, 33]. Very recently, we reported on the stability of the nitrogen mustard and the ester moieties in compounds **2–7** against hydrolysis and enzymatic cleavage in buffer, in the presence of porcine butyrylcholine esterase as well as in human and murine plasma [34]. The moderately basic morpholinoethyl ester (**4**) proved to be of particular interest with respect to both solubility and stability [34]. Preliminary data suggested considerably increased cytotoxicity of the esters compared to the parent compound **1**, the basic compounds being of particular interest [31]. Based on the assumption that higher antiproliferative activity may result from increased cellular accumulation, additional mechanisms of action or both, we compared compounds **2–7** with bendamustine (**1**) regarding cytotoxicity against a panel of human cancer cell types, representing hematologic and solid malignancies. Additionally, the induction of p53 expression and apoptosis, cellular enrichment and the involvement of the organic cation transporters OCT1 and OCT3 were investigated.

## Materials and Methods

### Ethics Statement

Human embryonal kidney (HEK293) cells were purchased from the German Collection of Microorganism and Cell Cultures (DSMZ, Braunschweig, Germany). Cancer cell lines were obtained from the American Type Culture Collection (ATCC; Rockville, MD, USA): SK-MEL-3 (malignant melanoma; HTB-69), Capan-1 (pancreatic adenocarcinoma; HTB-79), HT-29 (colorectal adenocarcinoma; HTB-38), SK-ES-1 (Ewing´s sarcoma; HTB-86), HEL92.1.7 (erythroleukemia; TIB-180), Jurkat (acute T-cell leukemia; TIB-152), U-937 (histiocytic lymphoma; CRL-1593.2), LNCaP (prostate cancer; CRL-1740) and NCI-H460 (large cell lung cancer; HTB-177), and MG-63 (osteosarcoma; CRL-1427) cells. HaCaT human skin keratinocyte cells (cryovial, 300493) were from Cell Line Service (CLS, Eppelheim, Germany).

### Chemicals and Reagents

Bendamustine hydrochloride (**1**) and the esters **2–7** were kindly provided by Arevipharma (Radebeul, Germany). Stock and working solutions of **1–7** were prepared in dimethylsulfoxide (DMSO). Thiazolyl blue tetrazolium bromide (MTT), umbelliferone, 4-[4-(dimethylamino)styryl]-N-methylpyridinium iodide (ASP^+^), penicillin-streptomycin and tetrapentyl ammonium (TPA) were from Sigma (Taufkirchen, Germany). Hygromycin B was from A.G. Scientific, (San Diego, CA, USA). Fetal calf serum (FCS) was from Biochrom (Berlin, Germany). A 1 mM stock solution of TPA was prepared in phosphate buffered saline (PBS). The 100 μM stock solution of ASP^+^ was prepared in PBS containing 10% DMSO. HPLC-grade acetonitrile was from Fisher Scientific (Schwerte, Germany). Demineralized water was prepared with a Milli-Q system (Merck; Schwalbach, Germany). If not otherwise stated, all other reagents were of analytical grade and purchased from Sigma (Taufkirchen, Germany).

### Cell Culture

#### Cancer and HaCaT cells

All media were from Sigma (Taufkirchen, Germany) and supplemented with 10% FCS. SK-MEL-3, Capan-1, HT-29 and SK-ES-1 cells were cultured in McCoy´s 5A medium, supplemented with 2.2 g/L NaHCO_3_ (Merck, Darmstadt, Germany). HEL92.1.7, Jurkat, U-937, LNCaP and NCI-H460 were cultured in RPMI-1640 containing 110 mg/L sodium pyruvate, 10 mM HEPES (both from Serva, Heidelberg, Germany) and 2.0 g/L NaHCO_3_. MG-63 cells were cultured in EMEM supplemented with 2.2 g/L NaHCO_3_. HaCaT cells were cultured in RPMI-1640, containing 110 mg/l sodium pyruvate, 10 mM HEPES, 2.0 g/l NaHCO_3_ and additional 2.5 g/L D-glucose. All cell types were passaged following treatment with a solution containing 0.05% trypsin and 0.025% EDTA. In case of human keratinocytes (HaCaT cells) cells were pre-incubated with 0.05% EDTA for 10 min at 37°C before trypsination.

#### Genetically modified HEK293 cells

Transfected human embryonic kidney cells (HEK293) were cultured in EMEM, supplemented with penicillin/streptomycin (100 U/mL / 100 μg/mL) and 250 μg/mL hygromycin B. The cells were cultured in a water-saturated atmosphere with 5% CO_2_ at 37°C and passaged once to twice a week. Adherently growing cells were treated with trypsin/EDTA (0.5 mg/mL / 0.22 mg/mL) (PAA, Pasching, Austria) and washed with medium prior to transfer into new culture flasks. Cells growing in suspension were passaged after mechanical separation of cell agglomerates.

#### HEK293 cells expressing the human organic cation transporters OCT1 or OCT3

Parental HEK293 cells were transfected with the plasmid pcDNA3.1/Hygro(-)-OCT1, containing the full length *SLC22A1* cDNA (NM_003057) encoding the human OCT1 protein, as described [35]. After hygromycin selection, single colonies were characterized for *SLC22A1* mRNA expression by real-time qRT-PCR, and the cell clone with the highest expression was further analyzed with respect to protein expression and [^3^H]-1-methyl-4-phenylpyridinium ([^3^H]MPP^+^) uptake (cf. Supporting Information). HEK-Co cells (control) were established by the same method using the empty plasmid pcDNA3.1/Hy(-) for transfection [36] (characterization of HEK-OCT1 and HEK-Co cells cf. S1 Methods; S1 Fig). HEK-OCT3 cells were recently described [37].

### Chemosensitivity Assays

Depending on the characteristics of the cells, two different assays were performed. For adherently growing cells (HaCaT, HT-29, NCI-H460, SK-MEL-3, MG-63, Capan-1), cytotoxic and cytocidal effects were determined in the crystal violet assay [38], whereas for loosely adherent cells (LNCaP, SK-ES-1) and cells growing in suspension (HEL 92.1.7, Jurkat, U-937) the MTT-assay [39] was used with minor modifications [38]. In brief, 1.5 · 10^3^ (HaCaT, HT-29, NCI-H460, SK-MEL-3, MG-63, Capan-1) or 5 · 10^3^ (Jurkat, U-937, HEL92.1.7, LNCaP, SK-ES-1) cells per well were seeded into flat-bottom 96-well plates (Sarstedt, Nümbrecht, Germany). All compounds were used as solutions prepared in DMSO and tested at final concentrations (8 replicates per concentration) of 1, 3, 10, 30 and 50 μM. In case of the vehicle control and and test compounds, the final DMSO concentration amounted to 0.1%. Absorbance was measured at 580 nm in a 96-well plate reader (GENios pro microplate reader, Tecan, Salzburg, Austria). Effects were quantified as previously described [40] for both assays.

In addition to long-term exposure, IC_50_ values were determined after an incubation period of 96 hours, following the same procedure as described above (4 instead of 8 replicates). As suggested by the National Cancer Institute [41] the corrected T/C values (T/C_corr_) were plotted against the logarithm of the concentrations, and the IC_50_ values were calculated using Prism 5.01 (GraphPad Software, La Jolla, CA, USA) according to the “*log (inhibitor) vs*. *normalized response-variable slope”* equation.

### Detection of Apoptosis (Annexin V/Propidium Iodide Assay)

Apoptosis was determined by incubating proliferating Jurkat cells with medium containing **1**, **2**, **4** or **5** at a concentration of 10 μM or 0.1% of DMSO (untreated control). After different periods of incubation (6, 24, 48 hours), samples were analyzed using the Annexin V-FITC apoptosis detection kit l (BD Biosciences, Heidelberg, Germany) according to the manufacturer’s protocol using 10^6^ cells/mL. Cells were analyzed using a FACSCalibur flow cytometer (BD Biosciences, Heidelberg, Germany). The compensation was performed for each experiment with annexin V-FITC (530/30 BP filter) and propidium iodide (585/42 BP filter), respectively. At least 1 · 10^4^ events were registered per sample and debris as well as cell aggregates were excluded by forward (FSC) versus side scatter (SSC) gates. Raw data were analyzed using FlowJo V10 software (Treestar Inc., Ashland, OR, USA).

### Detection of p53 Expression by Immunoblotting

The expression of the tumor suppressor p53 by NCI-H460 and HT-29 cells was determined after incubating the cells with compounds **1, 2, 4** and **5** at different concentrations for 24 hours. The cells (from a 10-cm culture dish, 70% confluency) were washed twice with PBS and harvested by scraping after addition of ice-cold buffer A (10 mM HEPES pH = 7.9, 10 mM KCl, 0.1 mM EDTA, 0.1 mM EGTA, protease inhibitor mix (Sigma-Aldrich)). After adding Nonidet P-40 (NP-40) (Sigma, Taufkirchen, Germany) to a final concentration of 1%, the cell suspensions were vortexed and subsequently centrifuged at 13000 g and 4°C for 30 seconds. The pellets were re-suspended in buffer B (buffer A + 400 mM NaCl, 1% NP-40) and gently agitated using a Sarmix M2000 (Sarstedt, Nümbrecht, Germany) at 4°C for 15 min, followed by centrifugation (13000 g, 4°C, 5 min; Microfuge; Eppendorf, Hamburg, Germany). The concentration of soluble protein was determined according to Bradford using the Bio-Rad Protein Assay (Bio-Rad Laboratories, Munich, Germany).

An amount of 30 μg total protein of each sample and 8 μL of a biotinylated molecular weight standard (1:6 dilution) (Cell Signaling, Danvers, MA, USA) was separated by SDS-PAGE (12% gel) and afterwards electroblotted (150 V, 30 min) to nitrocellulose membrane (Peqlab, Erlangen, Germany). To prevent unspecific binding, the membranes were treated with milk powder (5% (m/v) in buffer (150 mM NaCl, 20 mM Tris, 0.1% Tween 20, pH = 7.6)) before incubation with anti-p53 rabbit mAb (dilution 1:1000) (Cell Signaling) and anti-histone H2B Ab (dilution 1:1000) (Cell Signaling) as loading control at 4°C for 14 hours. The primary antibodies and the “protein ladder” were simultaneously detected with a horseradish peroxidase (HRP)-coupled donkey anti rabbit mAb (dilution 1:1000) (Santa Cruz biotechnology, Heidelberg, Germany) or the anti-biotin HRP-coupled antibody (dilution 1:5000) (Cell Signaling), respectively. The incubation with the secondary antibody was performed at room temperature for 1 hour. The bands were detected by bioluminescence using the Pierce ECL Western Blotting Substrate (Thermo Scientific, Dreieich, Germany). After exposure, the x-ray film (Amersham Hyperfilm ECL, GE Healthcare, München, Germany) was developed (CAWOMAT 2000 IR, CAWO, Schrobenhausen, Germany) and analyzed using a GS-710 imaging densitometer and Quantity One V. 4 software (Bio-Rad Laboratories, Munich, Germany).

### Quantification of Cell-Associated Bendamustine and Derivatives

Accumulation of bendamustine and selected bendamustine esters by HT-29 and NCI-H460 cells was determined by HPLC. For this purpose, 1.5 · 10^6^ cells/well were seeded into 6-well plates (Sarstedt, Nümbrecht, Germany). After two days of cultivation, the cells were incubated in PBS containing 30 μM of **1**, **2**, **4** or **5** and 200 μM of umbelliferone as internal standard at 37°C for 10 minutes. After removing the medium, the cells were washed three times with PBS. Untreated cells were detached with trypsin/EDTA and counted. The treated cells were harvested by adding 200 μL of ice-cold perchloric acid (1 M) and scraping. Subsequently, the samples were vortexed and sonicated (Branson 3200 ultrasonic cleaner; Branson, Danbury, USA) for 10 minutes. After 5 minutes of centrifugation (13000 g, 4°C), the supernatants were filtered (0.2 μm Phenex; Phenomenex, Aschaffenburg, Germany) and directly analyzed by HPLC with fluorescence detection according to a recently reported validated procedure [34]. The normalization of the measured concentration to the cell count allowed for a calculation of the cell-associated amount of the respective test compound. Additionally, the ratio of cell-associated substance compared to the applied concentration (30 μM) was calculated based on an average cell volume of 3 pL.

### Flow Cytometric Determination of OCT1 and OCT3 Activity

The previously described fluorescent substrate of organic cation transporters, 4-(4-dimethylaminostyryl)-N-methylpyridinium (ASP^+^) [42–45], was used to determine the function of OCT1 and OCT3 by flow cytometry in the absence and the presence of bendamustine and selected bendamustine esters. HEK293 cells expressing the transporter of interest were trypsinized and washed twice with PBS, prior to re-suspension of 0.5 · 10^6^ cells in 500 μL of PBS containing 2% (v/v) FCS. The cells were incubated with ASP^+^ (100 μM stock solution in PBS containing 10% DMSO) for 5 minutes, allowing for cellular accumulation, and subsequently analyzed with a FACSCalibur flow cytometer. ASP^+^ was excited at 488 nm and the fluorescence was measured using a 530/30 nm and a 585/42 band-pass filter. At least 1 · 10^4^ single cells were analyzed by appropriate FSC/SSC gating.

For the determination of K_m_, cell suspensions of HEK-OCT1 and HEK-OCT3 cells as well as HEK-Co cells as control for unspecific uptake of ASP^+^ were incubated with ASP^+^ at increasing concentrations at 37°C for 5 minutes. Subsequently, two volumes of an ice-cold 200 μM solution of the standard inhibitor of OCT1 and OCT3, tetrapentyl ammonium (TPA) [46–48], in PBS/FCS were added to one volume of cell suspension. The mixture was immediately analyzed. The mean fluorescence intensities (MFI) were calculated using FlowJo V10, and the difference between total (HEK-OCT1 or HEK-OCT3 cells) and unspecific (HEK-Co cells) uptake was plotted as specific uptake against the concentration of ASP^+^. The linearity of ASP^+^ uptake (1 μM) at 37°C was assessed between 30 seconds and 7 minutes (HEK-OCT1) or 9 minutes (HEK-OCT3), respectively. K_m_ was calculated according to the Michaelis-Menten equation using GraphPad Prism 5.01.

The inhibition of ASP^+^ uptake was measured to determine the IC_50_ values of TPA and compounds **1**, **2**, **4** and **5**. The cells were pre-incubated with the respective test compound at different concentrations at room temperature for 10 minutes. Afterwards, ASP^+^ was added to a final concentration of 1 μM, and the samples were incubated at 37°C in the dark for 5 minutes. Subsequently, the cells were washed, re-suspended in ice-cold PBS/FCS and stored on ice in the dark until measurement.

The normalized mean fluorescence intensities of the OCT expressing cells, set to 100% in the absence of an inhibitor, were calculated by subtracting the mean fluorescence intensity of unspecific ASP^+^ uptake into HEK-Co control cells. The normalized fluorescence intensities in the presence of an inhibitor were plotted against the logarithm of the concentrations of the test compounds to calculate IC_50_ values. The “*log (inhibitor) vs*. *normalized response-variable slope”* equation of GraphPad Prism 5.01 was used for this purpose.

### Imaging of Cellular ASP^+^ Uptake by Confocal Laser Scanning Microscopy

HEK-Co, HEK-OCT1 and HEK-OCT3 cells were seeded into 8-well μ-Slides (Ibidi, Munich, Germany) (2 · 10^4^ cells/well) and allowed to attach for 48 hours. Prior to the staining procedure, the medium was replaced by PBS and the DNA probe Draq5 (5 μM) (Biostatus, Shepshed, UK) was added to each well. To monitor the inhibition of ASP^+^ uptake, 200 μM of TPA or 15 μM of compound **5**, respectively, were added and incubated at 37°C for 5 minutes. Subsequently, ASP^+^ was added to reach a concentration of 1 μM, and the cells were incubated for 5 minutes in the dark. Imaging was performed with an Axiovert 200 M confocal microscope coupled to a Zeiss LSM 510 scanning device (Carl Zeiss, Oberkochen, Germany) using a Plan-Apochromat 63x/1.40 oil immersion objective. Draq5 was excited at 633 nm and fluorescence was detected using a 650 nm long-pass filter, ASP^+^ was excited at 488 nm and detected using a 565/35 nm band-pass filter.

### Determination of OCT1 and OCT3 Expression by Various Cancer Cells

The expression of *SLC22A1* and *SLC22A3* mRNA in different cancer cell types was analyzed by quantitative RT-PCR using the Roche LightCycler system. Total RNA was isolated using the NucleoSpin RNA Purification Kit (Macherey-Nagel, Dueren, Germany) according to the manufacturer’s instructions. One μg of total RNA was reversely transcribed using the iScript Kit (Biorad, Munich, Germany) according to the manufacturer´s instructions. *SLC22A1*, *SLC22A3* and *β-actin* mRNA levels were determined using the LightCycler System and the FastStart DNA Master SYBR Green I Kit (both from Roche, Mannheim, Germany). The primer pair for the amplification of the *SLC22A1* cDNA fragment was oOCT1-RT.for (CTGCCTGGTGAATGCTGAGC) and oOCT1-RT.rev (ACATCTCTCTCAGGTGCCCG), for the *SLC22A3* cDNA fragment oOCT3-RT.for (CAAGCAATATAGTGGCAGGGG) and oOCT3-RT.rev (CCTCAAAGGTGAGAGCGGGA) and for the *β-actin* fragment oActin.for (TGACGGGGTCACCACACACTGTGTGCCCATCTA) and oActin.rev (CTAGAAGCATTTGCGGTGGACGATGGAGGG). PCR was performed according to the manufacturer´s instructions with 0.5 μM of the respective sense and antisense primers, 4 mM MgCl_2_ and 1-fold FastStart DNA Master SYBR green I mix in a total volume of 20 μL including 1 μL of the synthesized sscDNA. Cycling conditions were as follows: 10 min denaturation at 94°C, followed by 45 cycles of 10 s denaturation at 94°C, 15 s primer annealing at 64°C and 30 s of elongation at 72°C. The amount of *β-actin*, *SLC22A1* and *SLC22A3* cDNAs were determined using a serial plasmid dilution (pOCT1.31; from 10^6^ to 10^4^ fg) as amplification standard. The *β-actin* concentration, calculated in relation to the standard curve, was set to 100% and the respective *SLC22A1* and *SLC22A3* mRNA values are given as a percentage of *β-actin* amplification.

## Results and Discussion

### Cytotoxicity of Bendamustine and Derivatives

**Fig 2:**
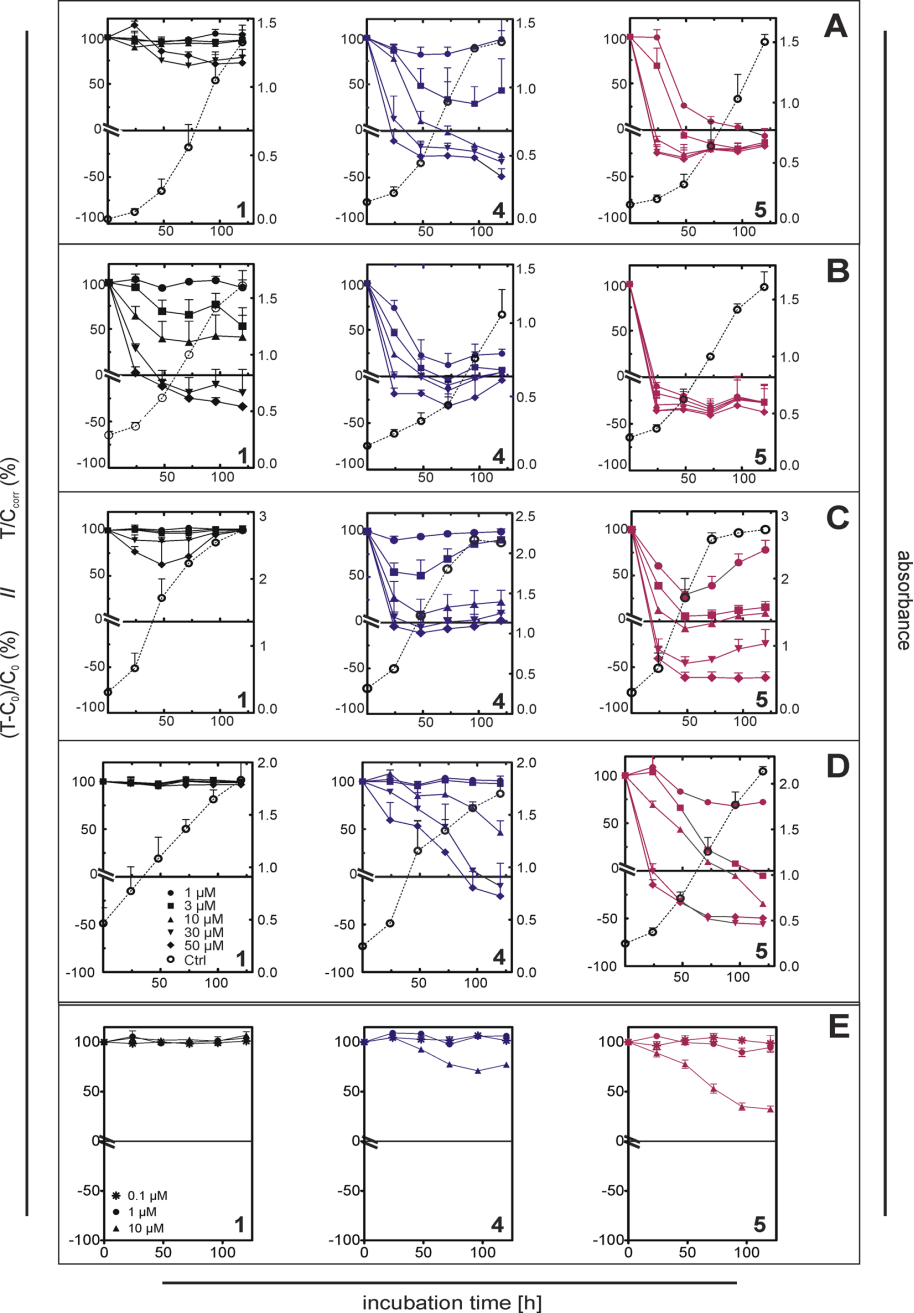
Antiproliferative activity of compounds 1, 4 and 5 against selected malignant tumor cells and spontaneously immortalized human keratinocytes upon long-term incubation. Jurkat (A; acute T cell leukemia), SK-ES-1 (B; Ewing’s sarcoma), NCI-H460 (C; large cell lung cancer) and HT-29 (D; colorectal cancer) cells were treated with compounds **1, 4** and **5** at concentrations between 1 μM and 50 μM, HaCaT cells (E; spontaneously immortalized human keratinocytes) were incubated with **1**, **4** and **5** at 0.1, 1 and 10 μM. Incubation period: 5 days. Antiproliferative and cytotoxic effects correspond to the left y-axes. The growth curves of untreated control cells (open circles) correspond to the right y-axes. Data are mean values ± SEM of at least 2 independent assays with 8 replicates per compound concentration. For additional data on long-term cytotoxity of compounds **1–7** against various tumor cell types and cytotoxicity of compound **2** against HaCaT cells cf. S3–S13 Figs.

The cytotoxicity of compounds **1–7** against tumor cells was determined both as an end point and kinetically. Additionally, the toxicity of **1**, **2**, **4** and **5** was determined in kinetic assays at spontaneously immortalized human keratinocytes (HaCaT), [49], as a model for “normal” cells. IC_50_ values of compounds **1–7** (Table 1 and S2 Fig) were calculated after 96 hours of incubation and the cytotoxic drug effect was measured over a period of 5 days (Fig 2 and S3–S13 Figs). In case of the crystal violet assay, the kinetic approach allows the distinction between cytotoxic, cytostatic and cytocidal drug effects [40].

**Table 1:** Cytotoxicity of compounds 1–7 against selected human cancer cell lines of different origin. **S**ubgroups, representing hematological malignancies, sarcoma, carcinoma and malignant melanoma, are separated by horizontal lines. IC_50_ values (μM) after 96 hours of incubation.

Cell line	Supporting information	Compound						
		1	2	3	4	5	6	7
**HEL 92.1.7** ^a^	S3 Fig	86.2 ± 2.6	2.5 ± 0.1	2.9 ± 0.2	2.4 ± 1.0	1.0 ± 0.01	1.3 ± 0.1	1.7 ± 0.1
**Jurkat** ^a^	S4 Fig	43.4 ± 4.8	3.1 ± 0.1	3.8 ± 0.04	3.2 ± 1.0	0.61 ± 0.02	0.83 ± 0.01	0.90 ± 0.03
**U-937** ^a^	S5 Fig	83.4 ± 8.5	1.8 ± 0.1	1.9 ± 0.01	3.2 ± 0.96	0.83 ± 0.19	1.1 ± 0.2	1.3 ± 0.3
**MG-63** ^b^	S6 Fig	55.8 ± 1.9	1.9 ± 0.1	2.1 ± 0.06	3.5 ± 0.1	0.38 ± 0.06	0.53 ± 0.01	0.55 ± 0.01
**SK-ES-1** ^a^	S7 Fig	9.6 ± 0.3	0.18 ± 0.02	0.18 ± 0.01	0.91 ± 0.08	0.09 ± 0.01	0.19 ± 0.01	0.19 ± 0.03
**Capan-1** ^b^	S8 Fig	18.9 ± 4.5	0.60 ± 0.04	1.0 ± 0.01	1.4 ± 0.01	0.12 ± 0.02	0.17 ± 0.01	0.25 ± 0.02
**LNCaP** ^a^	S9 Fig	77.9 ± 5.6	0.95 ± 0.15	1.5 ± 0.1	2.2 ± 0.1	0.50 ± 0.08	0.75 ± 0.03	0.93 ± 0.12
**NCI-H460** ^b^	S10 Fig	90.0 ± 4.3	4.8 ± 0.3	4.8 ± 0.1	9.7 ± 1.3	0.90 ± 0.01	1.2 ± 0.1	1.2 ± 0.1
**HT-29** ^b^	S11 Fig	> 100 ^c^	9.4 ± 0.4	10.0 ± 0.10	9.0 ± 0.5	1.1 ± 0.1	1.7 ± 0.1	2.4 ± 0.1
**SK-MEL-3** ^b^	S12 Fig	> 100 ^c^	13.8 ± 0.4	21.7 ± 4.10	20.9 ± 0.9	0.83 ± 0.18	1.24 ± 0.1	1.0 ± 0.27

Data are mean values ± SEM of 3 independent assays with 4 replicates per compound concentration. For cytotoxicity data after long-term exposure (5 days) cf. Fig 2 and S3–S12 Figs as indicated.

^a)^ MTT-assay

^b)^ Crystal violet assay

^c)^ Mean value ± SEM of 2 independent assays

Although **1** is approved for hematologic malignancies, the effects on representative tumor entities were rather weak (Table 1, Fig 2 and Supporting Information S3–S5 Figs). The IC_50_ values were > 80 μM against HEL 92.1.7 (erythroleukemia) and U-937 (histiocytic lymphoma), and 43 μM against Jurkat cells (acute T-cell leukemia, Fig 2A). By contrast, the Ewing´s sarcoma cells SK-ES-1 showed a distinct response upon treatment with bendamustine (IC_50_ ~ 10 μM; Table 1, Fig 2B), whereas the chemosensitivity of MG-63 osteosarcoma cells was markedly lower (IC_50_ ~ 56 μM) with cytocidal drug effects at concentrations above 30 μM (S6 Fig). Surprisingly, with an IC_50_ value < 20 μM Capan-1 pancreatic cancer cells showed moderate chemosensitivity. On the contrary, **1** was ineffective (SK-MEL-3 melanoma, HT-29 colorectal carcinoma) or only very weakly active (LNCaP prostate cancer; NCI-H460 large cell lung cancer) against the other carcinoma cell types (IC_50_ values 78 to > 100 μM).

In contrast to the parent compound bendamustine, the derivatives **2–7** exhibited considerably higher potencies up to factors > 100 both, against the cancer cells investigated. With respect to the cytotoxic effects, compounds **2–7** fall into two groups: the alkyl esters (**2, 3**) and the mofetil ester **4** on one hand, and the basic heterocyclic esters **5–7** on the other hand. Compared to bendamustine, compounds **2–4** showed a 10- to 30-fold increase in potency, and **5–7** were 60- to 120-fold more potent than **1**.

**Compounds 2–4:** Among compounds **2–4**, minor differences were observed at SK-ES-1 and NCI-H460 cells (Table 1) with a more pronounced effect of the alkyl esters **2** and **3** against SK-ES-1 cells (IC_50_ values: **2** and **3**: ~ 0.2 μM, **4**: 0.9 μM). The increase in toxicity compared to **1** was most striking at HEL92.1.7, LNCaP, MG-63 and, in case of the alkyl esters **2** and **3**, at SK-ES-1 cells. Comparable IC_50_ values of **2–4** were confirmed by the cytotoxic drug effects over a period of 5 days (S3–S13 Figs).

**Compounds 5–7:** Except for the hematologic malignancies (HEL92.1.7, Jurkat, U-937), SK-ES-1 and LNCaP cells, compounds **5–7** exhibited a distinct increase in cytotoxicity compared to **2–4**, showing IC_50_ values in low micro- to nanomolar range and cytocidal effects at concentrations as low as 1–10 μM at all treated cancer cells. The antiproliferative activity of compounds **5–7** was comparable both, in the end-point (cf. Table 1) and in the kinetic assays over a period of 5 days (S3–S12 Figs). Remarkably, compared to **1**, compounds **5–7** were up to > 100 times more potent against tumor cells such as SK-MEL3, NCI-H460, HT-29 and MG-63, which were refractory against treatment with bendamustine.

The results of the kinetic toxicity assays on HaCaT cells (Fig 2, S13 Fig) compared to the IC_50_ values (Table 1) and the data from kinetic cytotoxicity assays on cancer cells (Fig 2 and S3–S13 Figs) revealed a preferential toxicity against tumor cells, suggesting a more favorable “therapeutic index”, in particular in case of the esters **4** and **5**.

Data on the in vitro cytotoxicity of bendamustine are scarce. In the literature, for myeloma cells IC_50_ values around 100 μM or even higher are reported [22, 23]. A very recent study on several hematologic malignancies revealed IC_50_ values between approximately 10 μM and 250 μM. Especially mantle cell lymphoma, Burkitt’s lymphoma and T-cell acute lymphoblastic leukemia derived cell lines were relatively sensitive to bendamustine treatment [50]. The IC_50_ value (approximately 50 μM) reported by Hiraoka et al. [50] for Jurkat cells is in good agreement with our data. Published plasma levels of **1** after intravenous administration (C_max_ = 6 μg/mL (≈ 17 μM) [51]; C_max_ = 11 μg/mL (≈ 31 μM) [52]) suggest that the chemotherapy with bendamustine must be considered ineffective in case of tumor entities showing IC_50_ values in the two- to three-digit micromolar range in vitro. In this context the up to 100-fold antiproliferative activity of the bendamustine esters, in particular **5–7**, suggest both, higher efficacy in case of malignancies for which the parent compound is approved and a possible extension of the scope of indications.

### Induction of Apoptosis and p53 Expression by Compounds 1, 2, 4 and 5

**Fig 3:**
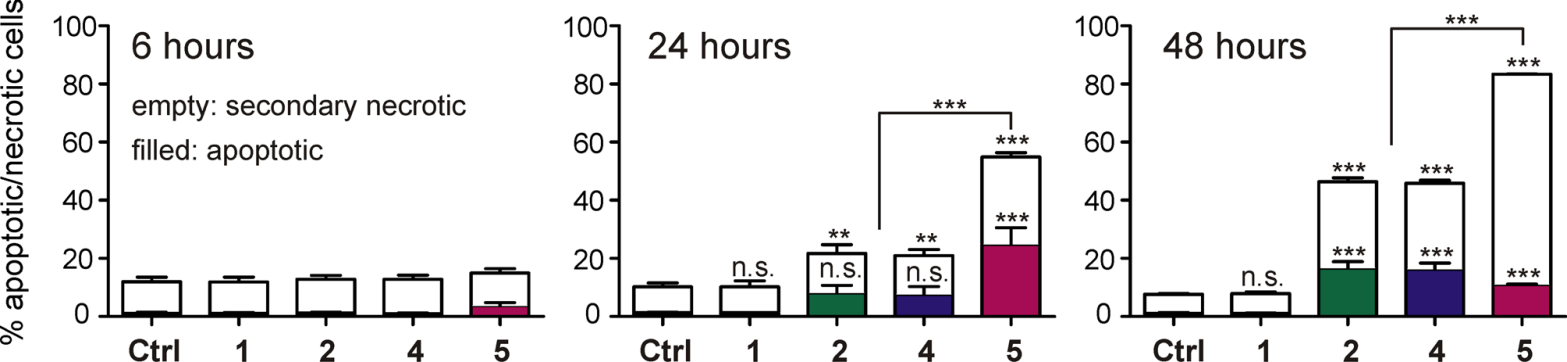
Induction of apoptosis in Jurkat cells. Results of annexin V/propidium iodide staining, performed after incubating Jurkat cells with compounds **1**, **2**, **4** and **5** at a concentration of 10 μM for different periods. Annexin V^+^/PI^-^ cells were defined as early apoptotic, whereas secondary apoptotic cells were annexin V^+^/PI^+^. Significances illustrated on top of the columns refer to the overall effect (apoptotic + secondary necrotic cells), those depicted in the columns refer to apoptotic cells. After 48 hours, apoptotic and secondary necrotic cells were significantly different (p < 0.001) (mean ± SEM, N = 3). One-way ANOVA and Bonferroni’s post-test were applied to calculate the significance of the apoptotic fraction and the sum of apoptotic and secondary necrotic cells; n.s.: not significant; *: p < 0.05; **: p < 0.01, ***: p < 0.001.

Bendamustine was reported to trigger apoptosis [22]. In search for an explanation of the higher antiproliferative activity of the bendamustine esters compared to the parent compound, we determined early and late stage of apoptosis by flow cytometry (annexin V/propidium iodide staining) after treatment of Jurkat cells with 10 μM of compounds **1, 2, 4** or **5** for 6, 24 and 48 hours (Fig 3; S14 Fig). As becomes obvious from Fig 3, annexin V^+^/PI^+^cells, defined as secondary necrotic cells, amounted to approximately 10% of the total cell population, most probably resulting from sample preparation. Only a fraction of approximately 1% of the cell population was Annexin V^-^/PI^+^ and considered necrotic. As expected from the results of the MTT assay, at a concentration of 10 μM, bendamustine had no effect compared to the control cells, regardless of the period of incubation. After 6 hours, the fraction of early apoptotic cells was less than 1% except for compound **5** (~2.5%; cf. Supporting Information, S14 Fig). Compounds **2** and **4** showed induction of apoptosis in around 10% of the cells after 24 hours of incubation. After 48 hours of incubation, 15% of the cells were in an early apoptotic state and a considerable fraction (20–25%) was secondary necrotic. In agreement with the results from the chemosensitivity assays, compound **5** was more potent, exhibiting a more rapid onset of action and a higher maximal response compared to **1**, **2** and **4** regarding induction of apoptosis. Approximately 50% of the cells were either early apoptotic (20%) or secondary necrotic (30%) after 24 hours. Two days after treatment, only around 15% of the cells were viable, whereas the majority of the cells was secondary necrotic (70%).

**Fig 4:**
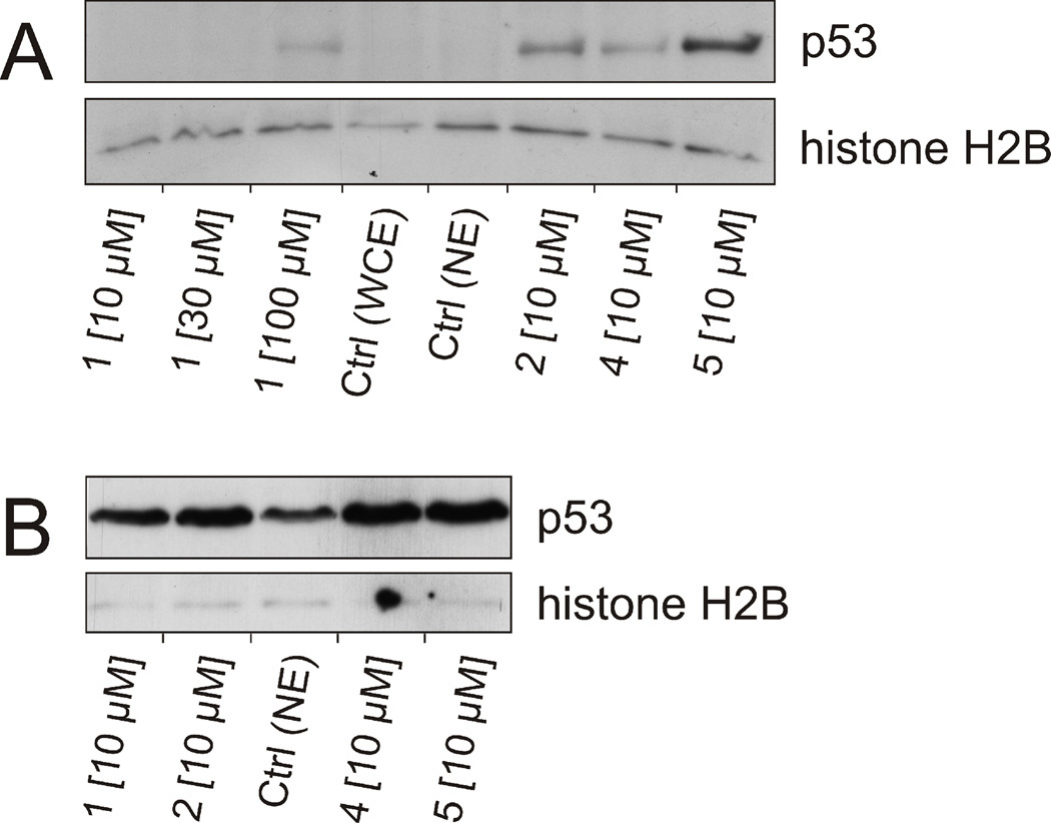
Effect of 1, 2, 4 and 5 on the expression of p53 in NCI-H460 and HT-29 cells. Western blot of p53 and histone H2B (loading control) of NCI-H460 (A) and HT-29 (B) cells. **A**: Nuclear extracts of NCI-H460 cells, incubated with different concentrations of **1** (10, 30 and 100 μM) or 10 μM of **2**, **4**, or **5** for 24 hours. Nuclear extracts (NE) and whole cell lysates (WCE) of untreated cells were used as control (Ctrl). **B**: Nuclear extracts of HT-29 cells, incubated with 10 μM of **1**, **2**, **4**, **5** for 24 hours. Untreated cells served as control (Ctrl).

Bendamustine was reported to induce the expression of p53 [21, 23, 50]. As the derivatives of **1** were much more potent against large cell lung and colorectal cancer cells, we investigated the induction of p53 expression in NCI-H460 and HT-29 cells after 24 hours of incubation with compounds **1, 2, 4** and **5** (Fig 4). Especially in NCI-H460 cells, p53 expression was significantly induced by the treatment with 10 μM of compounds **2**, **4** and **5**, while **5** led to the most pronounced effect. Bendamustine also weakly induced the expression of p53, although at a tenfold higher concentration (100 μM). Interestingly, p53 was only detectable in nuclear extracts of NCI-H460 cells after treatment, whereas HT-29 cells exhibited a high constitutive expression of the tumor suppressor protein. Nevertheless, the treatment with compounds **1**, **2**, **4** and **5** increased the expression of p53 in HT-29 cells but, similar to NCI-H460 cells, compounds **2**, **4** and **5** caused a higher expression level than **1**. Another notable observation was a slight induction of p53 expression in HT-29 cells after treatment with 10 μM of **1**, since proliferation assays revealed no toxic effect at this concentration. It is known from the literature that concentrations of ≥ 80 μM of bendamustine are required to produce a significant induction of p53 expression in various tumor cell types [22]. Both, the induction of p53 and apoptosis, correlated with the cytotoxic potency of bendamustine and derivatives.

### Cellular Accumulation of Bendamustine and Derivatives

**Fig 5:**
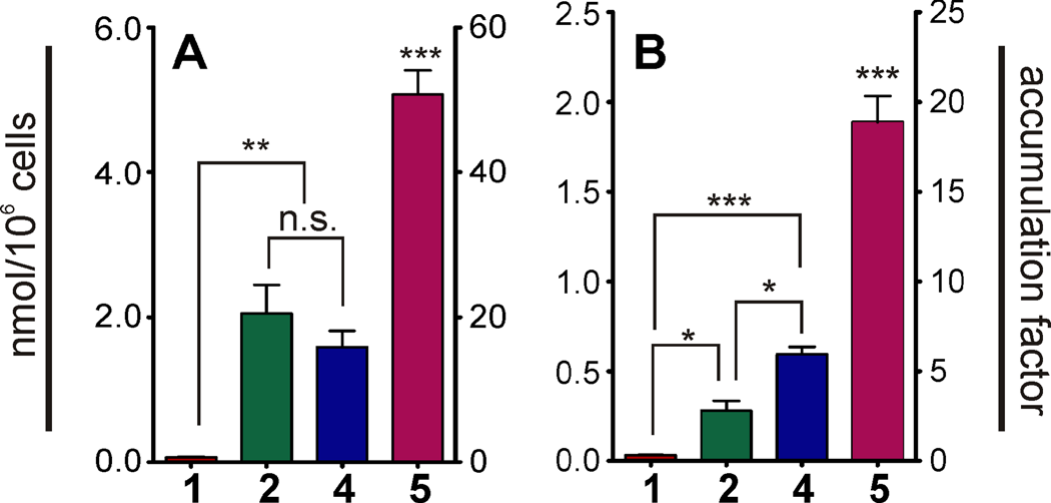
Accumulation of 1 and derivatives by HT-29 and NCI-H460 cells. Amount (mean ± SEM, N = 3–4) of cell-associated **1**, **2**, **4** and **5**, expressed as nmol/10^6^ cells (left y-axis) and cellular enrichment (right y-axis) in HT-29 (A) and NCI-H460 (B) cells. A mean cell volume of 3 pL was used to calculate accumulation factors. Significance was calculated using one-way Anova: n.s.: not significant; *: p < 0.05; **: p < 0.01, ***: p < 0.001.

Higher cellular uptake of the neutral (**2**, **3**) and basic (**4–7**) bendamustine esters compared to the parent compound could account for the increased potency in terms of antiproliferative activity, induction of apoptosis and p53 expression. Therefore, we performed HPLC analyses to determine the amount of cell-associated test compounds **1**, **2**, **4** and **5** at a concentration of 30 μM after 10 minutes of incubation. NCI-H460 and HT-29 cells were selected as examples of solid tumors, which surprisingly proved to be sensitive against bendamustine esters (Fig 5).

The recently reported validated HPLC method was applied [34]. To prevent hydrolysis of the nitrogen mustard group during sample preparation and analysis, cell lysis and deproteination were performed under acidic conditions (1 M perchloric acid, sonication). The esters were proven to be stable over the incubation period of 10 minutes in the presence of cells (S15 Fig). The cellular association of the internal standard umbelliferone did not significantly differ between both cell lines (p > 0.05). The amounts of cell-associated bendamustine (**1**) were extremely low (HT-29: 0.07 ± 0.011 nmol/10^6^ cells; NCI-H460: 0.03 ± 0.001 nmol/10^6^ cells). The ratio of cell-associated to the applied concentration of the test compound (accumulation factor) was 1:3 for NCI-H460 cells and 2:3 for HT-29 cells, indicating an incomplete uptake. In contrast, compounds **2**, **4** and **5** revealed considerably higher cell-associated amounts, with **5** reaching the highest cellular concentrations. A pronounced cellular enrichment of **2**, **4** and **5** was particularly observed in HT-29 cells. Compounds **2** (2.05 ± 0.39 nmol/10^6^ cells) and **4** (1.59 ± 0.23 nmol/10^6^ cells) revealed a 20- to 30-fold, and **5** (5.01 ± 0.34 nmol/10^6^ cells) an approximately 70-fold higher cellular enrichment than bendamustine. Qualitatively, the cellular accumulation of the bendamustine derivatives was comparable in HT-29 and NCI-H460 cells, though at a lower level (factor of approximately three) in case of the latter. The amounts of cell-associated **2**, **4** and **5** were 0.28 ± 0.06 nmol/10^6^ cells, 0.59 ± 0.04 nmol/10^6^ cells and 1.89 ± 0.14 nmol/10^6^ cells, respectively.

The levels of cellular enrichment correlate very well with the antiproliferative activities, underlining a crucial role of the ester moiety depending on the chemical nature, covering a neutral group (**2**) or substructures with different degree of basicity (**4** and **5**). In particular, the contribution of the pyrrolidino group in **5**, which is positively charged under assay conditions, becomes obvious from the increased cellular accumulation which is paralleled by the antiproliferative activity, the induction of apoptosis and p53 expression. The high cellular accumulation of **5** and the comparable effects of **5–7** concerning the toxicity suggest an important role of the basic substituent and the positive charge for the cellular association and thus for the toxicity. Apart from that, the significant difference between HT-29 and NCI-H460 cells might result from a different extent of diffusion and transporter-mediated uptake. Therefore, a possible contribution of organic cation transporters (OCT) was taken into account [53]. To test this hypothesis, functional studies on recombinant OCT1 and OCT3, expressed in HEK293 cells, were performed. Additionally, the expression of the respective transporters by the tumor cell types selected for cytotoxicity studies was investigated.

### Effect of Bendamustine Derivatives on the Activities of OCT1 and OCT3

#### ASP^+^ uptake by OCT1 and OCT3 expressing HEK293 cells

**Fig 6:**
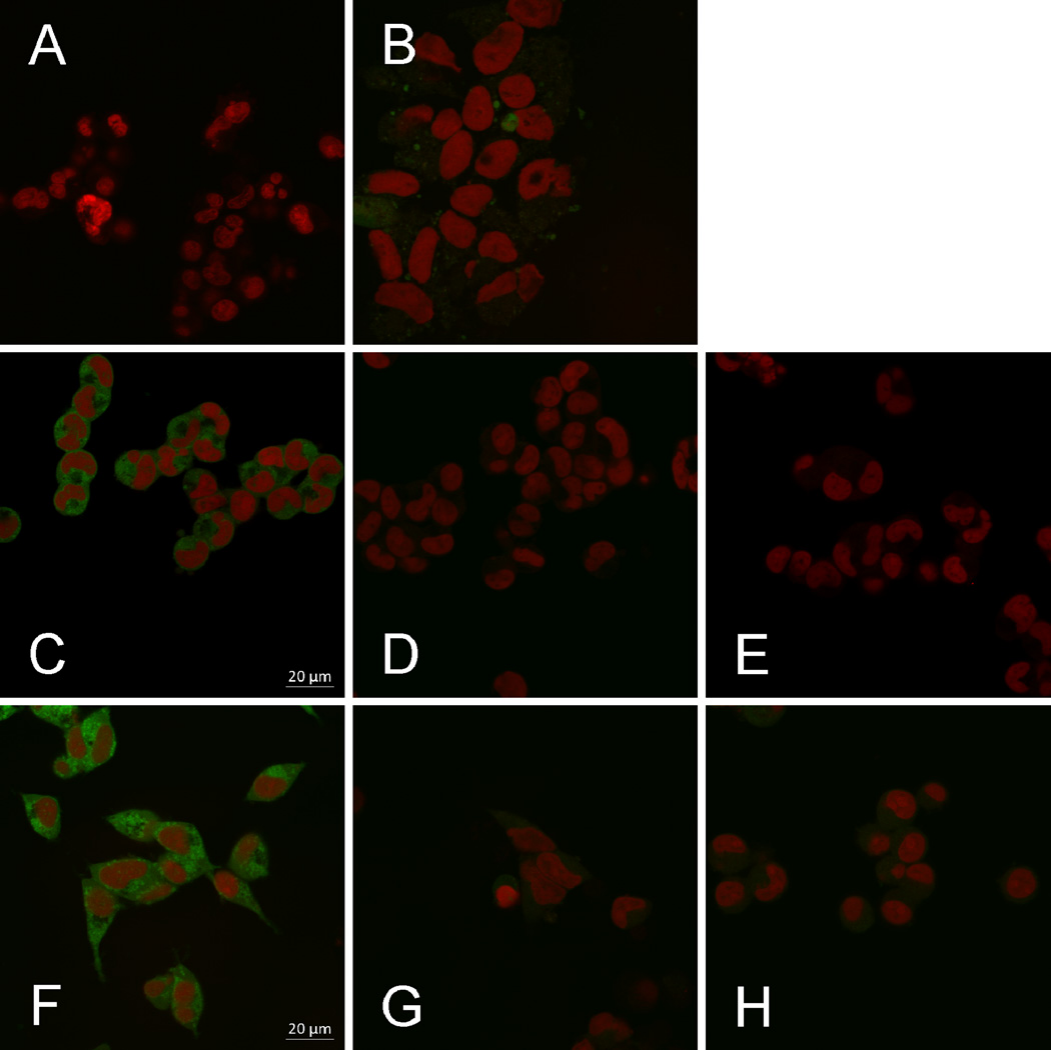
Confocal laser scanning microscopy of cellular ASP^+^ uptake via OCT1 and OCT3. Uptake of fluorescent ASP^+^ [1 μM] (green) in the absence and presence of compound **5** or TPA after 5 minutes of pre-incubation. Nuclei were stained with Draq5 [5 μM] (red). A, B: HEK-Co (control) cells, treated with Draq5 (A) or ASP^+^ plus Draq5 (B). C-E: HEK-OCT1 cells, treated with ASP^+^ plus Draq5 (C), ASP plus Draq5 plus compound **5** [15 μM] (D), or ASP^+^ plus Draq5 plus TPA [200 μM] (E). F-H: HEK-OCT3 cells, treated with ASP^+^ plus Draq5 (F), ASP^+^ plus Draq5 plus compound **5** [15 μM] (G), or ASP^+^ plus Draq5 plus TPA [200 μM] (H).

Confocal laser scanning microscopy revealed specific uptake of the fluorescent substrate ASP^+^ by organic cation transporter expressing HEK293 cells (Fig 6). OCT1 activity was lower than that of OCT3. The activities of both transporters were inhibited by compound **5** and the reference inhibitor TPA (Fig 6).

#### Determination of the affinities of OCT1 and OCT3 to ASP^+^ as substrate

**Fig 7:**
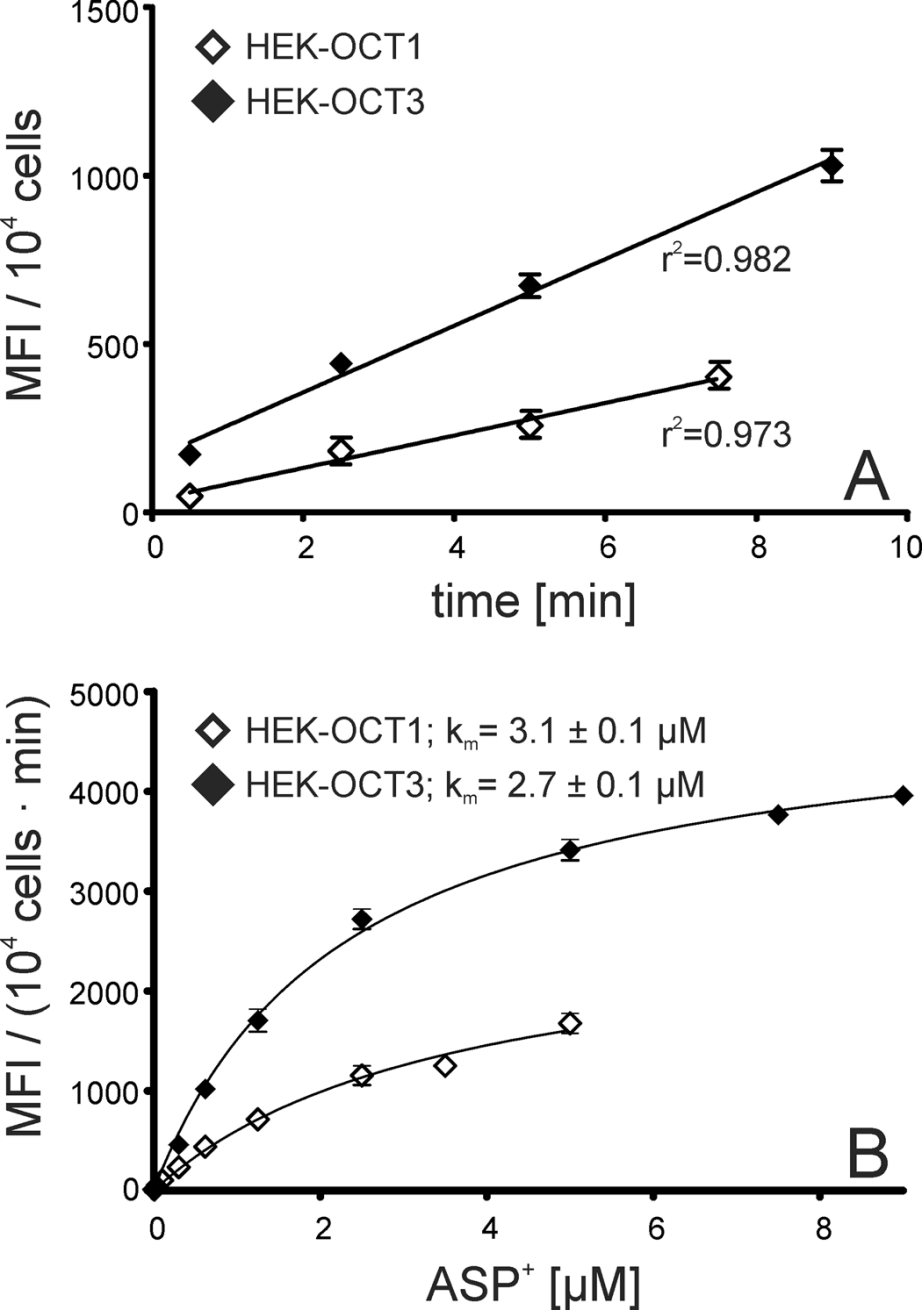
Specific uptake of ASP^+^ and Michaelis-Menten kinetics determined by flow cytometry. A: Time dependency of the specific mean fluorescence intensity (MFI) after incubating HEK-OCT1 and HEK-OCT3 cells with 1 μM ASP^+^ (means ± SEM, N = 3). B: Concentration dependency of the initial velocity of the specific increase in the mean fluorescence intensity (MFI) caused by ASP^+^-uptake into HEK-OCT1 and HEK-OCT3 cells. HEK cells transfected with an empty vector served as control for unspecific uptake (means ± SEM, N = 3).

Flow cytometry was applied to determine K_m_ values (Fig 7). The kinetics of ASP^+^-uptake and thus the increase in fluorescence were linear for both transporters (Fig 7A), allowing the determination of initial velocities (v_0_) at different concentrations (Fig 7B). The affinity to ASP^+^ was not significantly different for OCT1 (K_m_ = 3.1 ± 0.1 μM) and OCT3 (K_m_ = 2.7 ± 0.1 μM). By contrast, the maximal uptake rate for ASP^+^ was significantly higher for OCT3 [v_max_ = 5321 ± 89 MFI/(10^4^ cells · min)] than for OCT1 [v_max_ = 2808 ± 78 MFI/(10^4^ cells · min)] (p < 0.001).

#### Inhibition of OCT mediated ASP^+^ uptake by bendamustine derivatives

**Fig 8:**
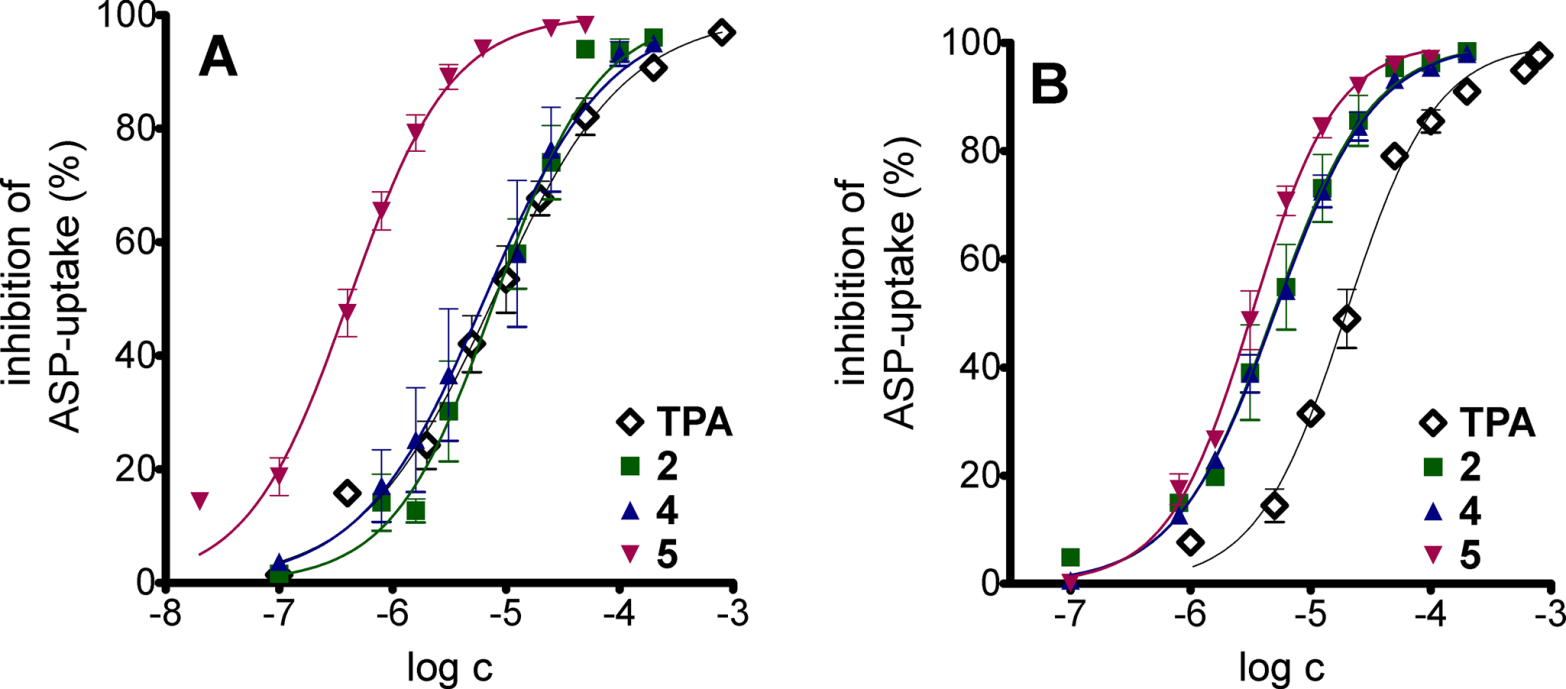
Concentration-dependent inhibition of ASP^+^-uptake by OCT expressing HEK-cells. Inhibition of ASP^+^-uptake (1 μM) into HEK-OCT1 (A) and HEK-OCT3 (B) cells by TPA, **2**, **4** or **5**. The mean fluorescence intensities were normalized to uninhibited ASP^+^-uptake (N = 3).

Bendamustine (**1**), the derivatives **2**, **4** and **5** and the standard inhibitor tetrapentyl ammonium (TPA) were investigated in the flow cytometric ASP^+^-uptake assay to determine IC_50_ values. The IC_50_ values of TPA in HEK-OCT1 (IC_50_ = 6.04 ± 0.83 μM) and HEK-OCT3 (IC_50_ = 19.9 ± 2.23) cells were consistent with previously published data, obtained in a microtiter assay (OCT1: IC_50_ = 7.9 μM [46], OCT3: IC_50_ = 28 μM [47]). Whereas compounds **2**, **4** and **5** (Fig 8) revealed concentration-dependent inhibition of both transporters, incomplete inhibition of OCT1 (35%) and OCT3 (37%) was observed at the highest examined concentration of bendamustine (200 μM). Compounds **2** and **4** were comparable to TPA at OCT1, whereas **5** was more potent by a factor of approximately 20 (Table 2). By contrast, compounds **2**, **4** and **5** were almost equipotent at OCT3 and approximately four times more potent than the reference compound TPA (p ≤ 0.05). Several studies conducted in the last years focused on the uptake of cytostatic drugs by organic cation transporters. Oxaliplatin [54], picoplatin [55], imatinib [56], paclitaxel and irinotecan [57] were identified as OCT1 substrates and oxaliplatin [58], melphalan, irinotecan and vincristine [59] as substrates for OCT3. Compared to other cytostatic drugs such as cisplatin (IC_50_ value > 100 μM at OCT1 [60]) and mitoxantrone (IC_50_ 16 μM at OCT1; 440 μM at OCT3 [61]), especially **5** revealed remarkably higher potency at OCT1 (IC_50_ 0.35 ± 0.03 μM). Whether compounds **2**, **4** and **5** are inhibitors or substrates of OCT1 and OCT3 is a matter of question. Supposed that these bendamustine derivatives, in particular the basic ester **5**, are substrates, accumulation and cytotoxicity should be related to the OCT expression.

**Table 2:** Inhibition of ASP^+^-uptake into HEK-OCT1 and HEK-OCT3 cells. IC_50_ (μM) values ± SEM (N = 3).

Compound	OCT1	OCT3
**TPA**	6.0 ± 0.8	19.9 ± 2.2
**1**	> 200	> 200
**2**	11.3 ± 1.9	5.1 ± 1.4
**4**	10.1 ± 2.5	5.1 ± 0.02
**5**	0.35 ± 0.03	3.41 ± 0.16

#### Expression of OCT1 (*SLC22A1*) and OCT3 (*SLC22A3*) by cancer cells

**Fig 9:**
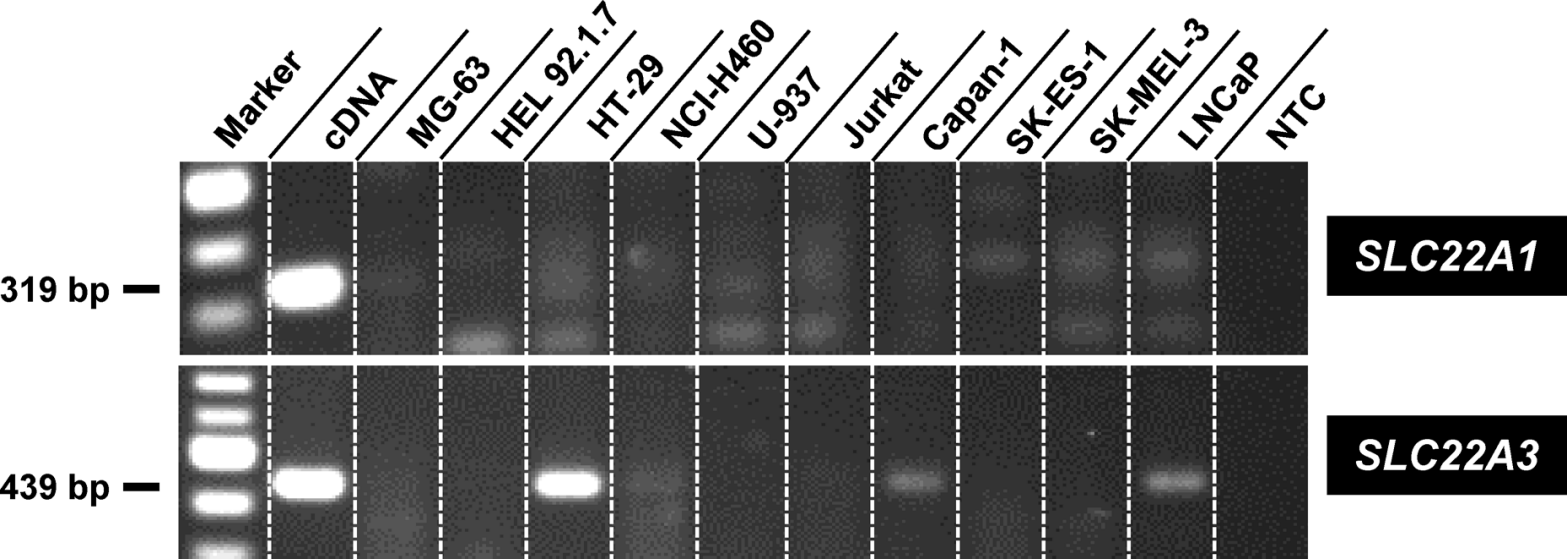
Expression of OCT1 and OCT3 by various cancer cells. Gel electrophoretic analysis of *SLC22A1* (encoding human OCT1) and *SLC22A3* (encoding human OCT3) mRNA expression by different human cancer cell types after RT-PCR. Human *SLC22A1* mRNA was detected using the primer pair oOCT1-RT.for–oOCT1-RT.rev, resulting in a specific band of 319 bp, human *SLC22A3* mRNA by the primer pair oOCT3-RT.for–oOCT3-RT.rev, resulting in a band of 439 bp. As a positive control, the plasmids pOCT1.31 and pOCT3.31 (cDNA) were used as templates of *SLC22A1* and *SLC22A3*, respectively. NTC = non-template control.

Previously, OCT1-mediated uptake of cytostatics in tumor cells was reported for chronic myeloid leukemia [56], chronic lymphocytic leukemia [57], and colon carcinoma [54], whereas OCT3 expression was associated with colorectal [58] and renal cancer [59]. We applied RT-PCR for the examination of OCT1 and OCT3 expression in all used cancer cell lines (Fig 9, S16 Fig). Whereas OCT1 was weakly expressed in SK-ES1 and SK-MEL3 cells (in both cell lines below 0.3%, related to β-actin expression) OCT3 expression was high in HT-29 cells (0.52%, proportional to β-actin expression), and clear bands were detectable upon analysis of Capan-1 (0.05%, proportional to β-actin expression) and LNCaP cells (0.14%, proportional to β-actin expression). The differential expression (Fig 9, S16 Fig) does not support the hypothesis that the chemosensivities of the investigated cancer cell types (Table 1) are primarily determined by OCT-mediated uptake of the bendamustine esters.

## Conclusion

Although the investigated compounds are sufficiently stable to act as antitumor agents on their own, it cannot be precluded that the esters are only prodrugs, allowing for increased intracellular accumulation of bendamustine. Having the N-Lost moiety in common, alkylating property is a characteristic feature of both, the parent compound and the derivatives. In concert with the increase in apoptotic processes and elevated p53 expression, the data may be interpreted as a hint to a dual mechanism of action, in particular in case of the basic bendamustine esters.

## Supporting Information

S1 Fig Characterization of HEK293 cells stably expressing human OCT1. A: Immunoblot analysis of lysates (20 μg each) of HEK-Co and HEK-OCT1 cells. OCT1 was detected with the antiserum KEN (see: Material and Methods). B: Immunolocalization of human OCT1 in HEK-Co and HEK-OCT1 cells by confocal microscopy. The OCT1 protein was detected in the plasma membrane of transfected HEK-OCT1 cells and no OCT1-specific staining was detectable in HEK-Co cells. C: Concentration dependent [^3^H]MPP^+^ uptake in HEK-Co and HEK-OCT1 cells (incubation time: 3 min). D: Time-dependent uptake of [^3^H]MPP^+^ (50 μM) by HEK-Co and HEK-OCT1 cells. In both cases OCT1-mediated uptake (squares) of [^3^H]MPP^+^ was determined by subtracting uptake in HEK-Co cells from the uptake into HEK-OCT1 cells.
(TIF)

S2 Fig Concentration-response curves of 1, 2 and 5 against Jurkat (A) and HT-29 (B) cells after 96 hours of incubation. T/C_corr_ values represent the net proliferation of the treated cells referred to the vehicle treated control cells (set to 100%). The obtained data were used for the calculation of IC_50_ values (mean values ± SEM of 2–3 independent experiments with 4 replicates per concentration).
(TIF)

S3 Fig Chemosensitivity of HEL92.1.7 cells against compounds 1 and 3–7. Antiproliferative/cytotoxic effects correspond to the left y-axes. The growth curves of untreated cells (open circles) correspond to the right y-axes. Mean values ± SEM of 2–3 independent assays with 8 replicates per concentration and time point.
(TIF)

S4 Fig Chemosensitivity of Jurkat cells against compounds 1 and 3–7. Antiproliferative/cytotocix effects correspond to the left y-axes. The growth curves of untreated cells (open circles) correspond to the right y-axes. Mean values ± SEM of 2–3 independent assays with 8 replicates per concentration and time point.
(TIF)

S5 Fig Chemosensitivity of U-937 cells against compounds 1 and 3–7. Antiproliferative/cytotoxic effects correspond to the left y-axes. The growth curves of untreated cells (open circles) correspond to the right y-axes. Mean values ± SEM of 2–3 independent assays with 8 replicates per concentration and time point.
(TIF)

S6 Fig Chemosensitivity of MG-63 cells against compounds 1 and 3–7. Antiproliferative and cytocidal effects correspond to the left y-axes. The growth curves of untreated cells (open circles) correspond to the right y-axes. Mean values ± SEM of 2–3 independent assays with 8 replicates per concentration and time point.
(TIF)

S7 Fig Chemosensitivity of SK-ES-1 cells against compounds 1 and 3–7. Antiproliferative/cytotoxic effects correspond to the left y-axes. The growth curves of untreated cells (open circles) correspond to the right y-axes. Mean values ± SEM of 2–3 independent assays with 8 replicates per concentration and time point.
(TIF)

S8 Fig Chemosensitivity of Capan-1 cells against compounds 1 and 3–7. Antiproliferative and cytocidal effects correspond to the left y-axes. The growth curves of untreated cells (open circles) correspond to the right y-axes. Mean values ± SEM of 2–3 independent assays with 8 replicates per concentration and time point.
(TIF)

S9 Fig Chemosensitivity of LNCaP cells against compounds 1 and 3–7. Antiproliferative/cytotoxic effects correspond to the left y-axes. The growth curves of untreated cells (open circles) correspond to the right y-axes. Mean values ± SEM of 2–3 independent assays with 8 replicates per concentration and time point.
(TIF)

S10 Fig Chemosensitivity of NCI-H460 cells against compounds 1 and 3–7. Antiproliferative and cytocidal effects correspond to the left y-axes. The growth curves of untreated cells (open circles) correspond to the right y-axes. Mean values ± SEM of 2–3 independent assays with 8 replicates per concentration and time point.
(TIF)

S11 Fig Chemosensitivity of HT-29 cells against compounds 1 and 3–7. Antiproliferative and cytocidal effects correspond to the left y-axes. The growth curves of untreated cells (open circles) correspond to the right y-axes. Mean values ± SEM of 2–3 independent assays with 8 replicates per concentration and time point.
(TIF)

S12 Fig Chemosensitivity of SK-MEL-3 cells against compounds 1 and 3–7. Antiproliferative and cytocidal effects correspond to the left y-axes. The growth curves of untreated cells (open circles) correspond to the right y-axes. Mean values ± SEM of 2–3 independent assays with 8 replicates per concentration and time point.
(TIF)

S13 Fig Chemosensitivity of NCI-H460 (A), HT-29 (B) and HaCaT (C) cells against compound 2. Antiproliferative and cytocidal effects correspond to the left y-axes. The growth curves of untreated cells (open circles) correspond to the right y-axes. Mean values ± SEM of 2–3 independent assays with 8 replicates per concentration and time point.
(TIF)

S14 Fig Flow cytometric analysis of Jurkat cells. Cells were treated with 10 μM **1**, **2**, **4** and **5** after different periods of incubation (6, 24, 48 hours) by annexin V-FITC (x-axis) and propidium iodide (y-axis) staining. Samples of untreated cells were collected at the same time points and served as control (**Ctrl**). The indicated percentages are related to the total number of collected single cells. The different test compounds are arranged horizontally.
(TIF)

S15 Fig Stability of compounds 1 and 4 in the presence of NCI-H460 cells. Representative chromatograms, indicating the stability of compounds **1** and **4** in the presence of NCI-H460 cells (IS = internal standard, umbelliferone). Samples were measured immediately before the incubation (t = 0 minutes) and after 10 minutes of incubation at 25°C (t = 10 minutes). The kinetics of the hydrolysis of the N-Lost group, yielding **1a** or **4a**, is the same in case of bendamustine (**1**) and the morpholinoethyl ester **4**, respectively. Other decomposition products were not detected. The same holds for compounds **2** and **5** upon incubation with cells under the same conditions (data not shown).
(TIF)

S16 Fig Semi-quantitative analysis of the *SLC22A1* and *SLC22A3* mRNA expression in different cancer cell types. *SLC22A1*, *SLC22A3* and *β-actin* mRNA levels were determined using the semiquantitative LightCycler system and the mRNA expression values of *SLC22A1* and *SLC22A3* are given in percentage of the *β-actin* amplification.
(TIF)

S1 Methods (PDF)
