# Supplementary figures and images for: Esters of Bendamustine Are by Far More Potent Cytotoxic Agents than the Parent Compound against Human Sarcoma and Carcinoma Cells
Source: PLoS One. 2015 Jul 21;10(7):e0133743. doi: 10.1371/journal.pone.0133743 (PMC4721923; doi:10.1371/journal.pone.0133743)

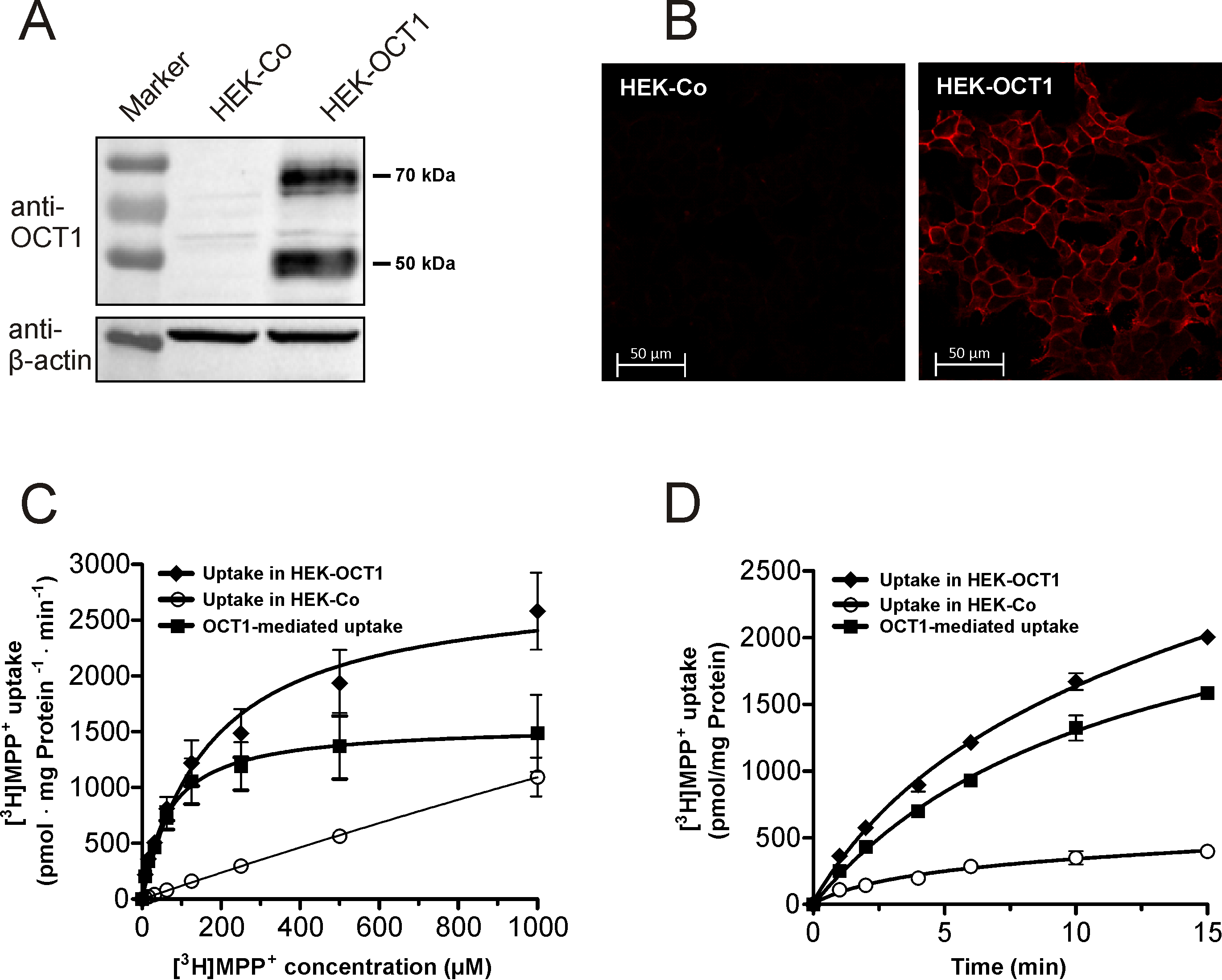

Supplement: S1 Fig — A: Immunoblot analysis of lysates (20 μg each) of HEK-Co and HEK-OCT1 cells. OCT1 was detected with the antiserum KEN (see: Material and Methods). B: Immunolocalization of human OCT1 in HEK-Co and HEK-OCT1 cells by confocal microscopy. The OCT1 protein was detected in the plasma membrane of transfected HEK-OCT1 cells and no OCT1-specific staining was detectable in HEK-Co cells. C: Concentration dependent [3H]MPP+ uptake in HEK-Co and HEK-OCT1 cells (incubation time: 3 min). D: Time-dependent uptake of [3H]MPP+ (50 μM) by HEK-Co and HEK-OCT1 cells. In both cases OCT1-mediated uptake (squares) of [3H]MPP+ was determined by subtracting uptake in HEK-Co cells from the uptake into HEK-OCT1 cells. (TIF) [file pone.0133743.s001.tif]

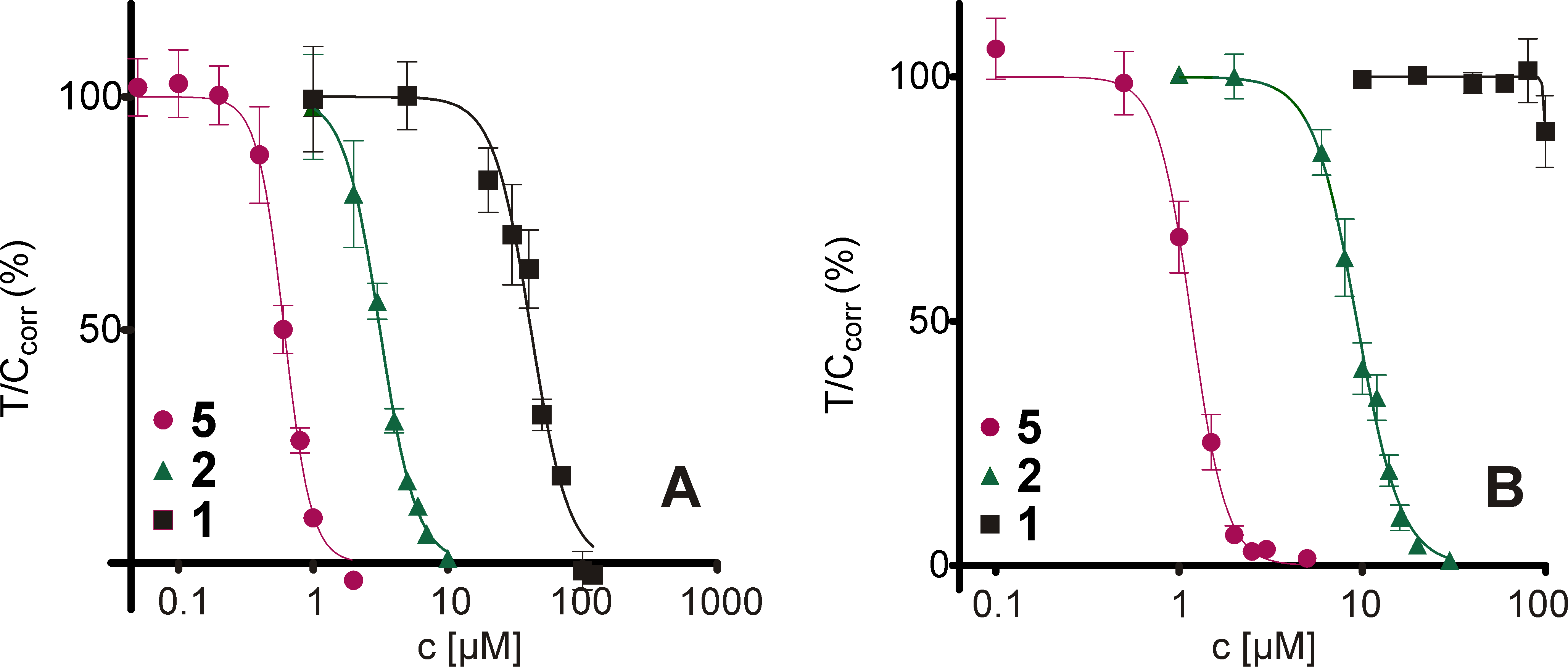

Supplement: S2 Fig — T/Ccorr values represent the net proliferation of the treated cells referred to the vehicle treated control cells (set to 100%). The obtained data were used for the calculation of IC50 values (mean values ± SEM of 2–3 independent experiments with 4 replicates per concentration). (TIF) [file pone.0133743.s002.tif]

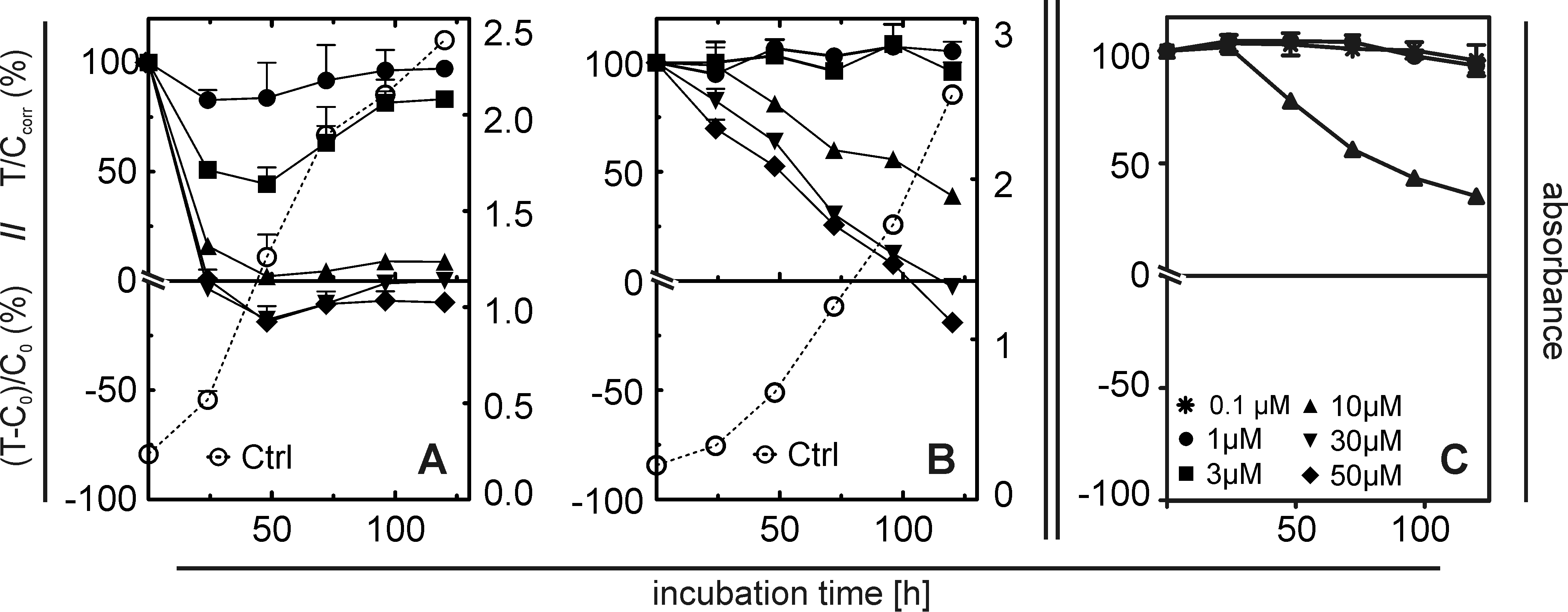

Supplement: S13 Fig — Antiproliferative and cytocidal effects correspond to the left y-axes. The growth curves of untreated cells (open circles) correspond to the right y-axes. Mean values ± SEM of 2–3 independent assays with 8 replicates per concentration and time point. (TIF) [file pone.0133743.s013.tif]

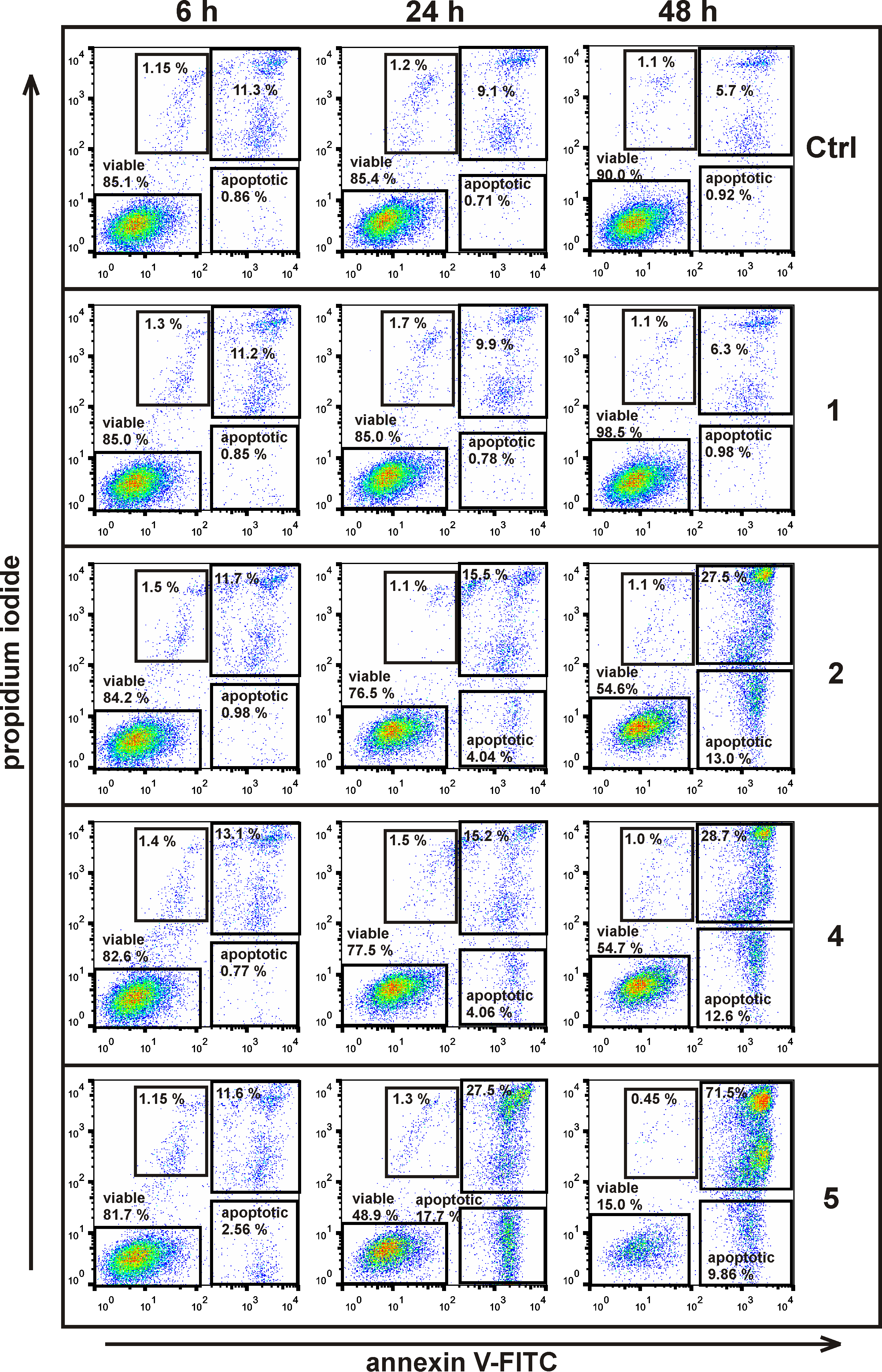

Supplement: S14 Fig — Cells were treated with 10 μM 1, 2, 4 and 5 after different periods of incubation (6, 24, 48 hours) by annexin V-FITC (x-axis) and propidium iodide (y-axis) staining. Samples of untreated cells were collected at the same time points and served as control (Ctrl). The indicated percentages are related to the total number of collected single cells. The different test compounds are arranged horizontally. (TIF) [file pone.0133743.s014.tif]

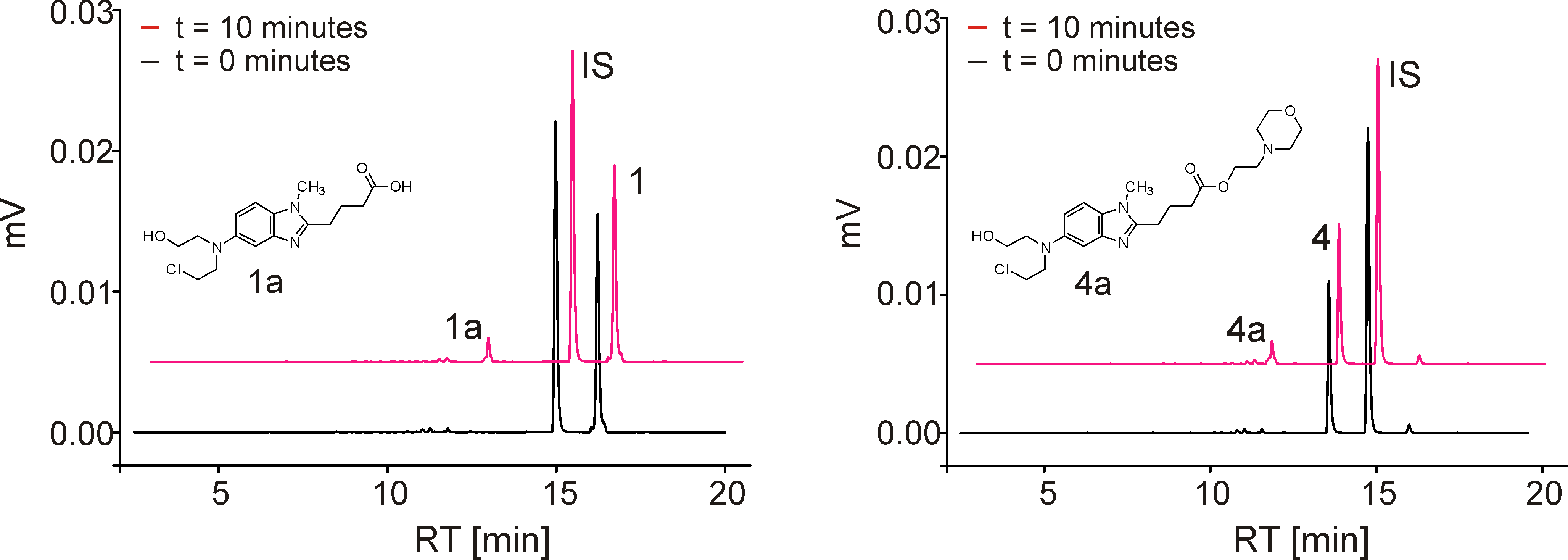

Supplement: S15 Fig — Representative chromatograms, indicating the stability of compounds 1 and 4 in the presence of NCI-H460 cells (IS = internal standard, umbelliferone). Samples were measured immediately before the incubation (t = 0 minutes) and after 10 minutes of incubation at 25°C (t = 10 minutes). The kinetics of the hydrolysis of the N-Lost group, yielding 1a or 4a, is the same in case of bendamustine (1) and the morpholinoethyl ester 4, respectively. Other decomposition products were not detected. The same holds for compounds 2 and 5 upon incubation with cells under the same conditions (data not shown). (TIF) [file pone.0133743.s015.tif]

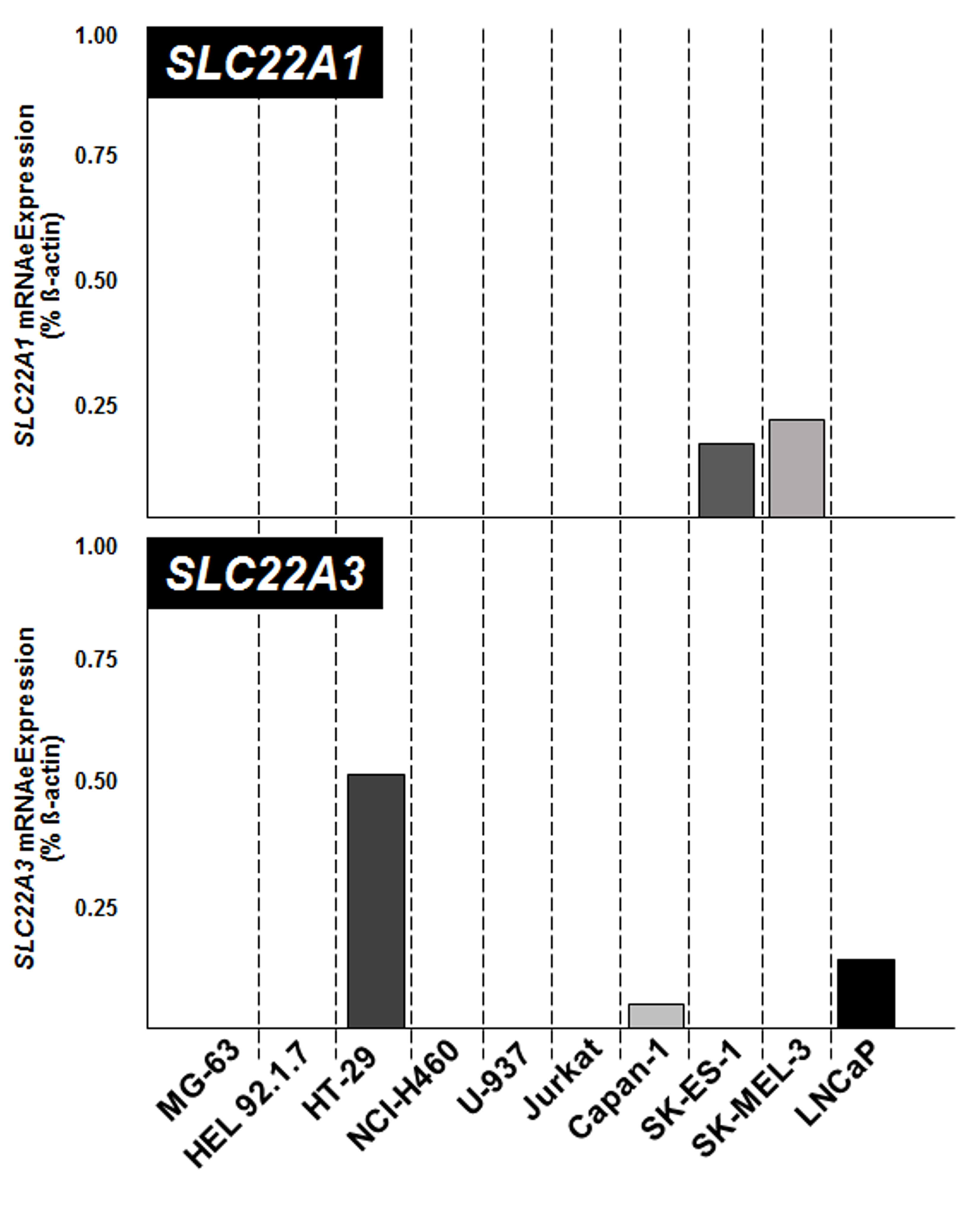

Supplement: S16 Fig — SLC22A1, SLC22A3 and β-actin mRNA levels were determined using the semiquantitative LightCycler system and the mRNA expression values of SLC22A1 and SLC22A3 are given in percentage of the β-actin amplification. (TIF) [file pone.0133743.s016.tif]
