# Supplementary material for: Esters of Bendamustine Are by Far More Potent Cytotoxic Agents than the Parent Compound against Human Sarcoma and Carcinoma Cells
Source: PLoS One. 2015 Jul 21;10(7):e0133743. doi: 10.1371/journal.pone.0133743 (PMC4721923; doi:10.1371/journal.pone.0133743)
Supplement: S1 Methods — (PDF) [file pone.0133743.s017.pdf]

Methods

Western blot analysis of hOCT1 expression

HEK-Co and HEK-OCT1 cells were lysed with 0.2% ice-cold SDS containing protease inhibitor mixture (Mini-complete protease inhibitor cocktail tablets; Roche Diagnostics-Applied Science, Mannheim, Germany). Protein concentration of each lysate was measured with a bicinchoninic acid assay (BCA protein assay kit, Thermo Fisher Scientific, Waltham, MA, USA). 20 µg of cell lysates were subjected to a 10% SDS-polyacrylamide gel electrophoresis under reducing conditions. Separated proteins were transferred onto nitrocellulose membranes (Protran nitrocellulose transfer membrane; Whatman, Dassel, Germany). Blots were probed with the KEN antiserum (1 : 10 000) directed against human OCT1 [1] overnight at 4 °C. Membranes were then incubated with a horseradish peroxidase-conjugated goat-anti-rabbit secondary antibody (1:10 000; Sigma Aldrich, Munich, Germany). Immunoreactive bands were visualized using an ECL western blotting detection reagent (GE Healthcare, Buckinghamshire, UK) and a Chemidoc XRS imaging system (Bio-Rad, Munich, Germany). To control sample loading, membranes were incubated for 30 min with restore western blot stripping buffer (Pierce, Rockford, USA) at 37 °C and reprobed with a mouse monoclonal anti-human β-actin antibody (1:10 000; Sigma Aldrich, Munich, Germany). A horseradish peroxidase-conjugated goat-anti-mouse antibody (GE Healthcare Europe, Freiburg, Germany) was used as secondary antibody (dilution 1:4 000).

Immunofluorescence detection of hOCT1 expression

The cellular localization of the OCT1 protein in the stably transfected HEK-OCT1 cells was analyzed by immunofluorescent staining. The OCT1-specific KEN antiserum was used at a dilution of 1:1 000. Subsequently, cells were incubated with an Alexa Fluor 488 conjugated goat-anti-rabbit secondary antibody (1:10,000; Invitrogen, Carlsbad, CA).

[^{3}H]MPP$^+$ transport assay

HEK-OCT1 and HEK-Co cells were seeded in poly-D-lysine (Sigma Aldrich, Taufkirchen, Germany) coated 48-well plates at a density of $1.2 \cdot 10^5$ cells/well. After incubation at 37 °C and 5% CO_2 for 48 h medium was replaced by warm (37 °C) uptake buffer (142 mM NaCl, 5 mM KCl, 1 mM K_2HPO_4, 1.2 mM $MgSO_4$, 1.5 mM $CaCl_2$, 5 mM glucose and 12.5 mM HEPES, pH 7.3). The uptake assay was started by addition of [^{3}H]MPP$^+$ (80 Ci/mmol, American Radiolabeled Chemicals, St Louis, MO) at indicated concentrations (concentration dependency) or at a concentration of 50 µM (time dependency). Cells were incubated at 37 °C for 3 min (concentration dependency) or at indicated time points (time dependency). Uptake was stopped by washing the cells three times with ice-cold uptake buffer. Afterwards, cells were lysed with 5 mM Tris buffer (pH 7.3) containing 0.1% Triton X-100. The intracellular accumulation of radioactivity was determined by liquid scintillation counting (PerkinElmer, Rodgau-Jügesheim, Germany) and protein concentration of each lysate was measured with the BCA assay. We performed two experiments each on

two different days ($n = 4$). The OCT1-mediated net uptake of [^{3}H]MPP^+ was determined as the difference in substrate uptake between HEK-OCT1 and HEK-Co cells. Data were presented as means ± standard error of the mean. A value of $p < 0.05$ was considered statistically significant.

References

1. Nies AT, Herrmann E, Brom M, Keppler D. Vectorial transport of the plant alkaloid berberine by double-transfected cells expressing the human organic cation transporter 1 (OCT1, SLC22A1) and the efflux pump MDR1 P-glycoprotein (ABCB1). Naunyn Schmiedebergs Arch Pharmacol. 2008;376: 449-461.
